# Supplementary material for: Substrate-interacting pore loops of two ATPase subunits determine the degradation efficiency of the 26S proteasome
Source: Nat Commun. 2026 Mar 24;17:4473. doi: 10.1038/s41467-026-70426-y (PMC13186983; doi:10.1038/s41467-026-70426-y)
Supplement: Supplementary file 1 — Supplementary Information [file 41467_2026_70426_MOESM1_ESM.pdf]

Supplementary Figures and Tables:

A

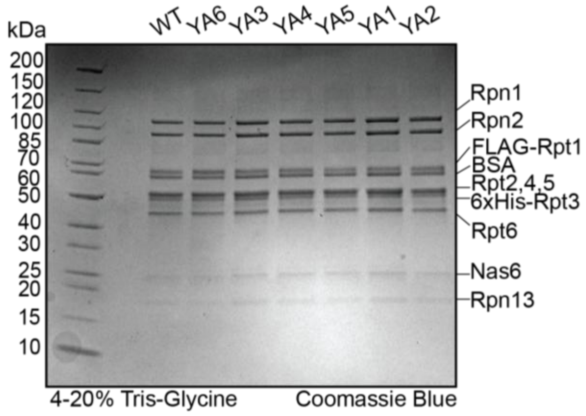

B

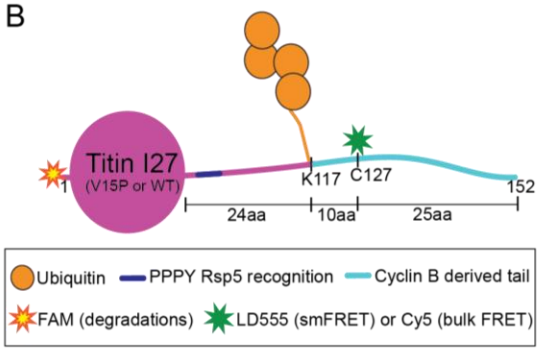

Variant	Amino acid sequence
Titin I27 V15P	MGGGLIEVERPLYGVEVFPGETAHFEIELSEPDVHGQWRL-RGQPLAASPDCEIIEDGRRHILILHNCQLGMTGEVSFQ AANTRSAANLRVRELGGAGPPPYSAANDENYALAAHG-GKHTFNNENVSCRLGGAASIAVQAPAQHTFNNENVSY
Titin I27 WT	MGGGLIEVERPLYGVEVFVGETAHFEIELSEPDVHGQWRL-RGQPLAASPDCEIIEDGRRHILILHNCQLGMTGEVSFQ AANTRSAANLRVRELGGAGPPPYSAANDENYALAAHG-GKHTFNNENVSCRLGGAASIAVQAPAQHTFNNENVSY

Supplementary Fig. 1: A) 4-20% Tris-Glycine SDS-PAGE of purified base subcomplexes (~3.2 µg), visualized by Coomassie blue stain. **B)** Titin I27 substrate used throughout this study, either without (wild type) or with a V15P destabilizing mutation as indicated in each assay. All lysine residues were removed from the folded domain, and a single lysine for ubiquitin attachment was introduced at position 117 within a C-terminally fused Cyclin B-derived unstructured tail (cyan). An Rsp5 E3 ligase recognition site (dark blue) was added to facilitate polyubiquitination (orange circles). In addition, the substrate was labeled either at the N-terminus through a Sortase A reaction with a FAM-LPETGG peptide, used to track substrate degradation, or at an engineered Cys127 with maleimide-linked SulfoCy5 or LD555 to follow substrate processing by FRET in bulk or at the single-molecule level, respectively. Sequences for the wild-type and V15P titin substrates are shown on the right.

A

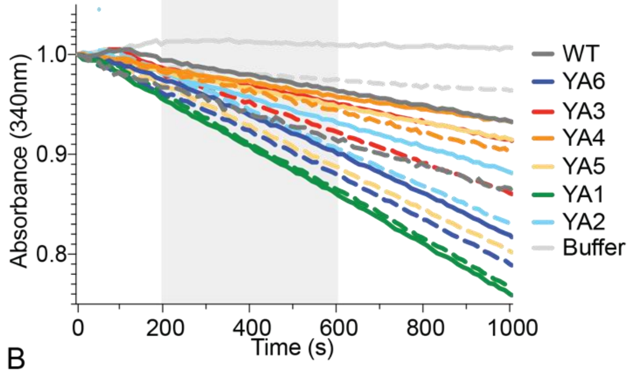

Variant	ATPase Rate (ATP enzyme^{-1} min^{-1})	
	Basal	+2uM ub'd Titin I27 ^{V15P}
WT	41 ± 1	98 ± 8
YA6	101 ± 2	113 ± 6
YA3	52 ± 4	95 ± 8
YA4	42 ± 2	85 ± 4
YA5	48 ± 1	122 ± 0
YA1	131 ± 6	137 ± 6
YA2	70 ± 2	116 ± 7

B

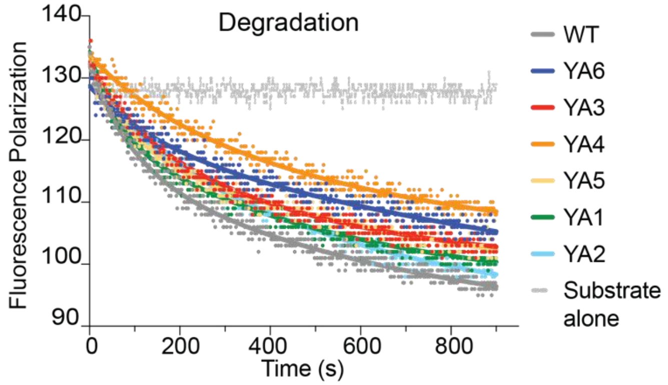

Variant	Titin I27 ^{V15P} Single Turnover Degradation Rate (substrate enzyme^{-1} min^{-1})
WT	0.81 ± 0.12
YA6	0.13 ± 0.00
YA3	0.54 ± 0.07
YA4	0.23 ± 0.06
YA5	0.59 ± 0.14
YA1	1.06 ± 0.07
YA2	0.73 ± 0.17

C

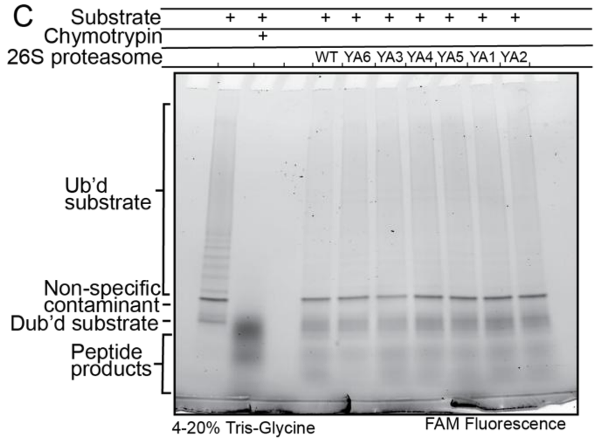

Supplementary Fig. 2: A) Representative traces for the ATP-hydrolysis measurement of *in vitro*-reconstituted proteasomes in the absence (dashed lines) or presence (solid lines) of 2 μM ubiquitinated FAM-titin I27^{V15P} in a NADH-coupled assay. The shaded area was used for linear regressions of the observed absorbance decays, with the resulting ATPase rates shown in the table on the right. Values represent the average of n = 3 technical replicates and SEM. **B)** Representative traces for the single-turnover degradation of the ubiquitinated FAM-Titin I27^{V15P}

substrate by *in vitro*-reconstituted proteasomes, monitored by the decrease in fluorescence polarization. Data were fit to a double exponential decay (solid line) using GraphPad Prism to obtain degradation rates shown in the table on the right. Values represent the average of n = 3 technical replicates and SEM. **C)** SDS-PAGE analysis of end-point samples for the single-turnover degradation of the ubiquitinated FAM-Titin I27^{V15P} substrate visualized using the FAM fluorescence emission channel.

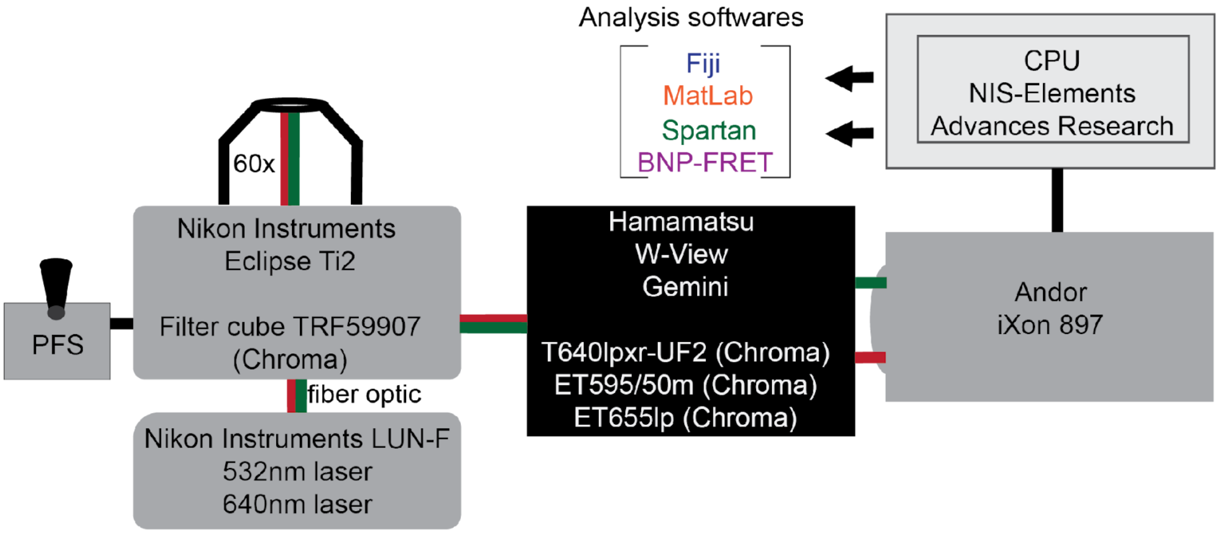

Supplementary Fig. 3: TIRF-microscope setup using the Nikon Instruments Eclipse Ti2, LUNF laser box, and Perfect Focus system, and the analysis softwares Fiji, MatLab, Spartan, and BNP-FRET.

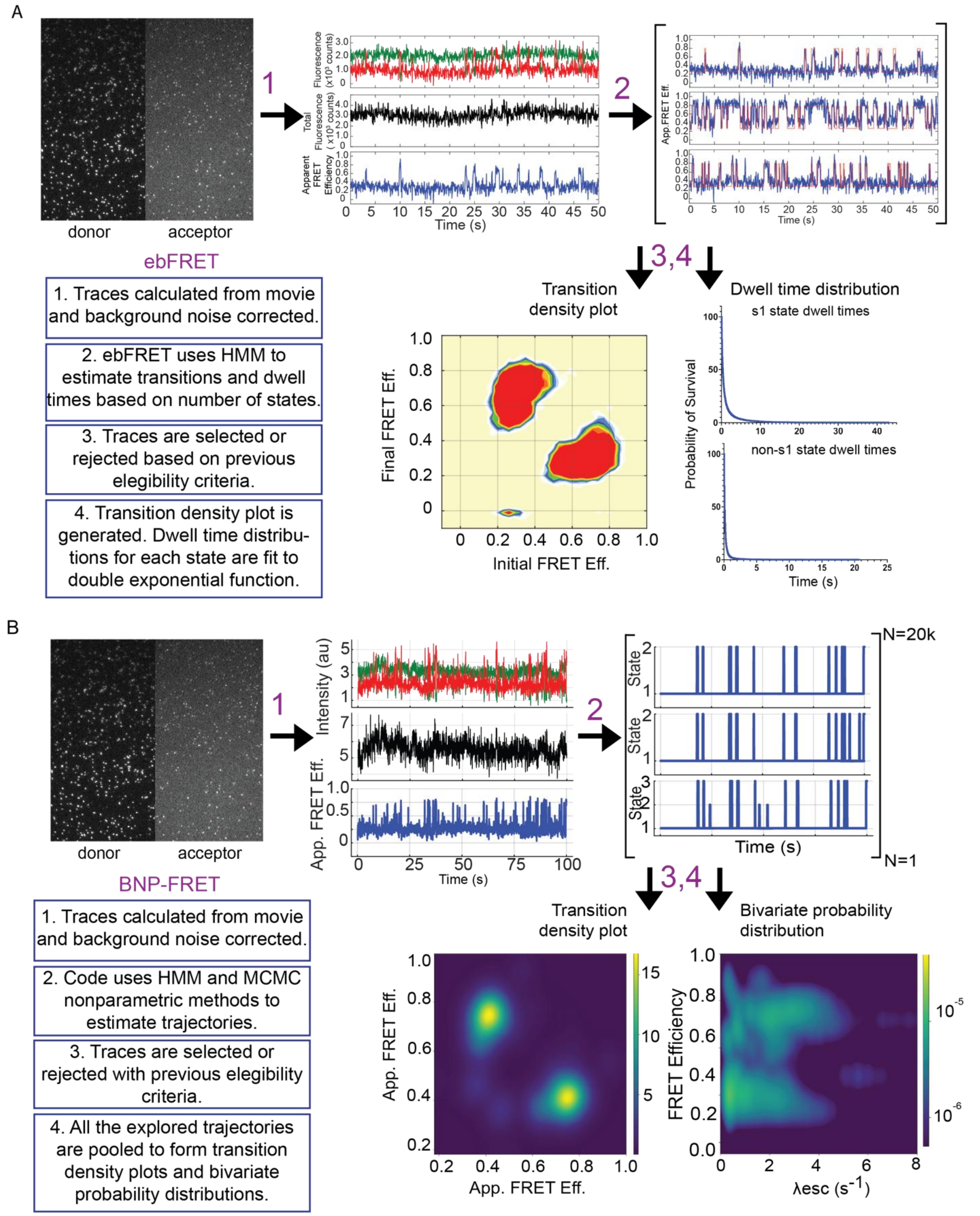

Supplementary Fig. 4: A) ebFRET Hidden Markov Model (HMM) and (B) BNP-FRET algorithm workflow for the analyses of the proteasome conformational dynamics.

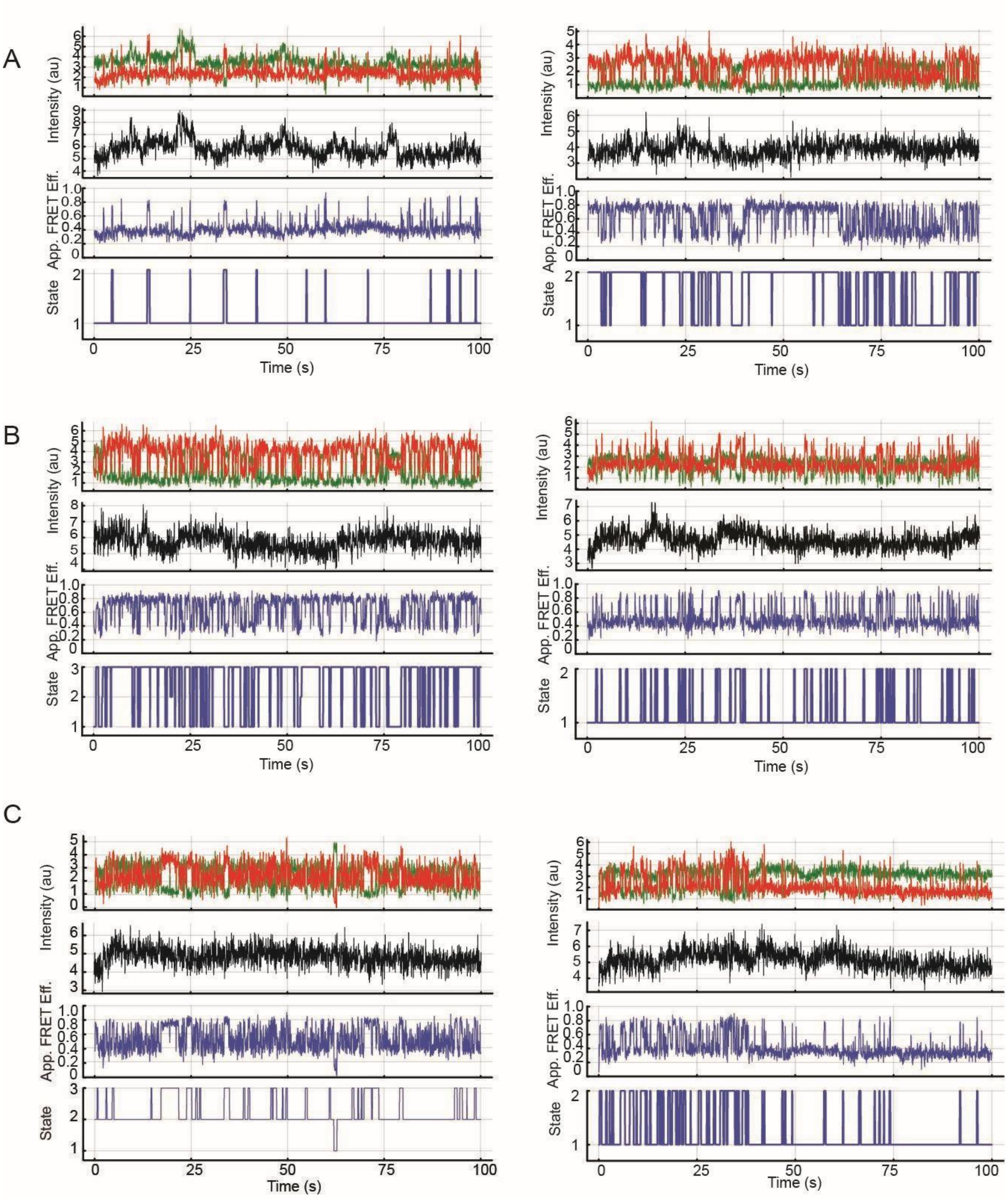

Supplementary Fig. 5. Representative traces from the conformational dynamics assay monitoring reconstituted wild-type **(A)**, YA6 **(B)**, and YA4-mutant proteasomes **(C)** in the absence of substrate. The first panels show fluorescence intensities for the FRET donor (green) and

acceptor (red), the second panels depict the total fluorescence intensities (black), the third panels show the apparent FRET efficiencies, and the fourth panels illustrate the estimated most-probable state trajectories as determined by the BNP-FRET algorithm.

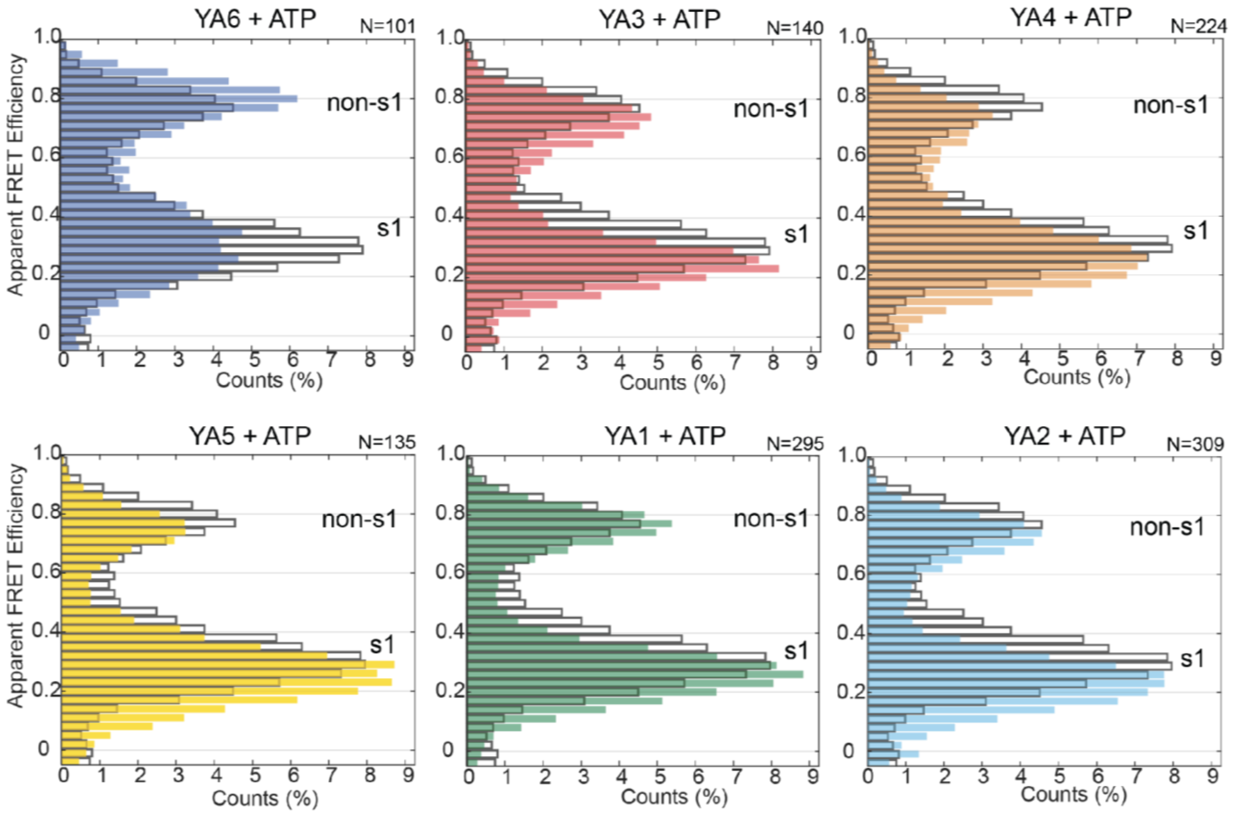

Supplementary Fig. 6. Apparent FRET efficiency distributions for YA6 (dark blue), YA3 (red),YA4 (orange), YA5 (yellow), YA1 (green), and YA2 (light blue) mutant proteasomes in ATP compared to the distribution of the wild-type proteasome (grey outlined bars).

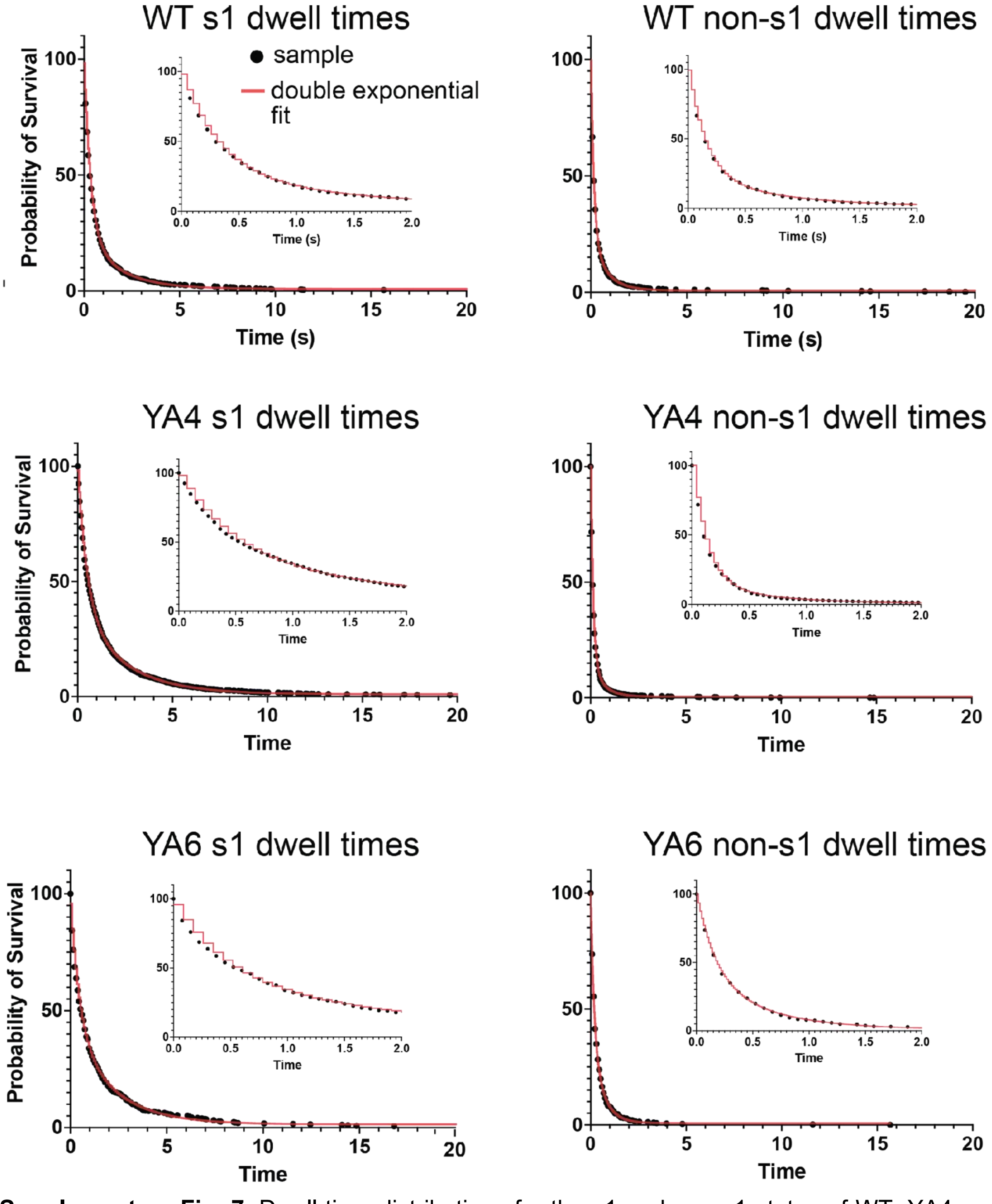

Supplementary Fig. 7. Dwell-time distributions for the s1 and non-s1 states of WT, YA4-, and YA6-mutant proteasomes in the presence of ATP from ebFRET analyses were fit to double exponentials (red lines) to determine the transition rates (see Fig. 3G,H). Insets show the zoom-ins for dwell times of 0 - 2 s.

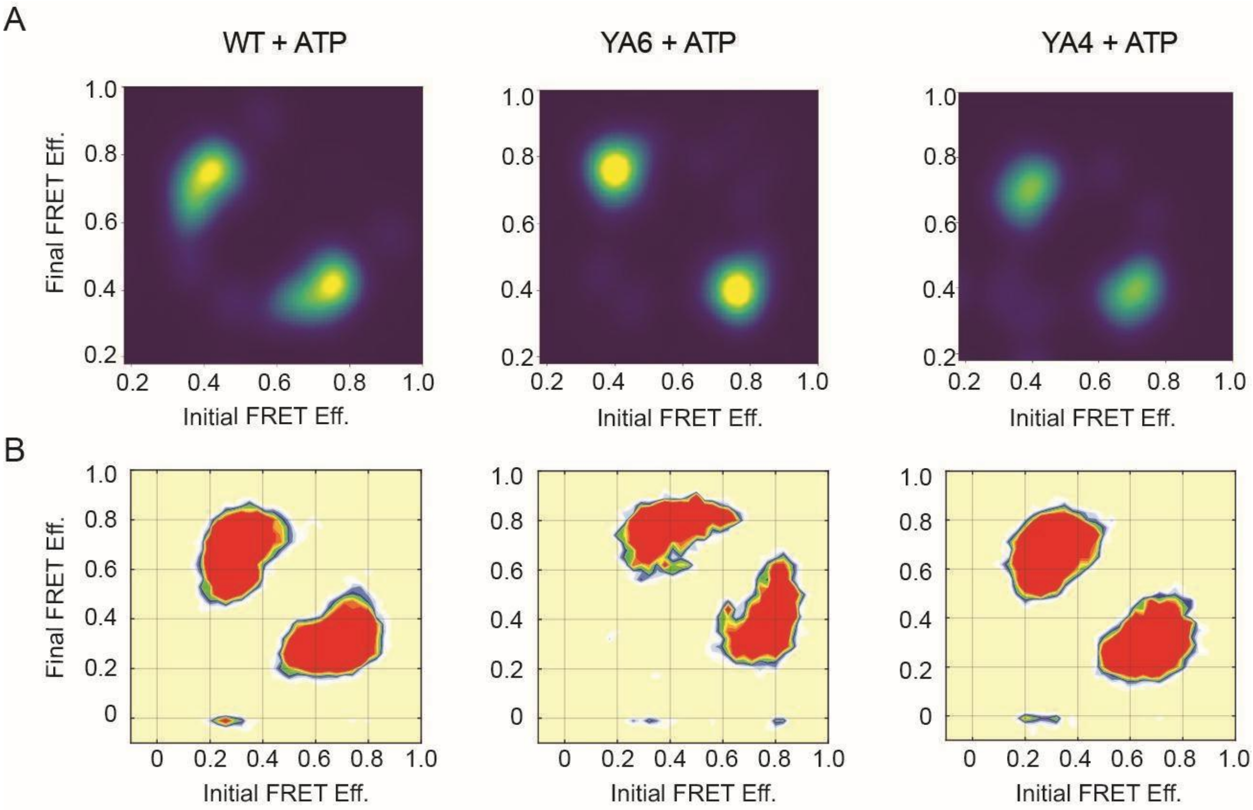

Supplementary Fig. 8. Transition density plots for wild-type, YA6, and YA4-mutant 26S proteasomes in the absence of substrate determined by the (A) BNP-FRET algorithm or (B) the Spartan software.

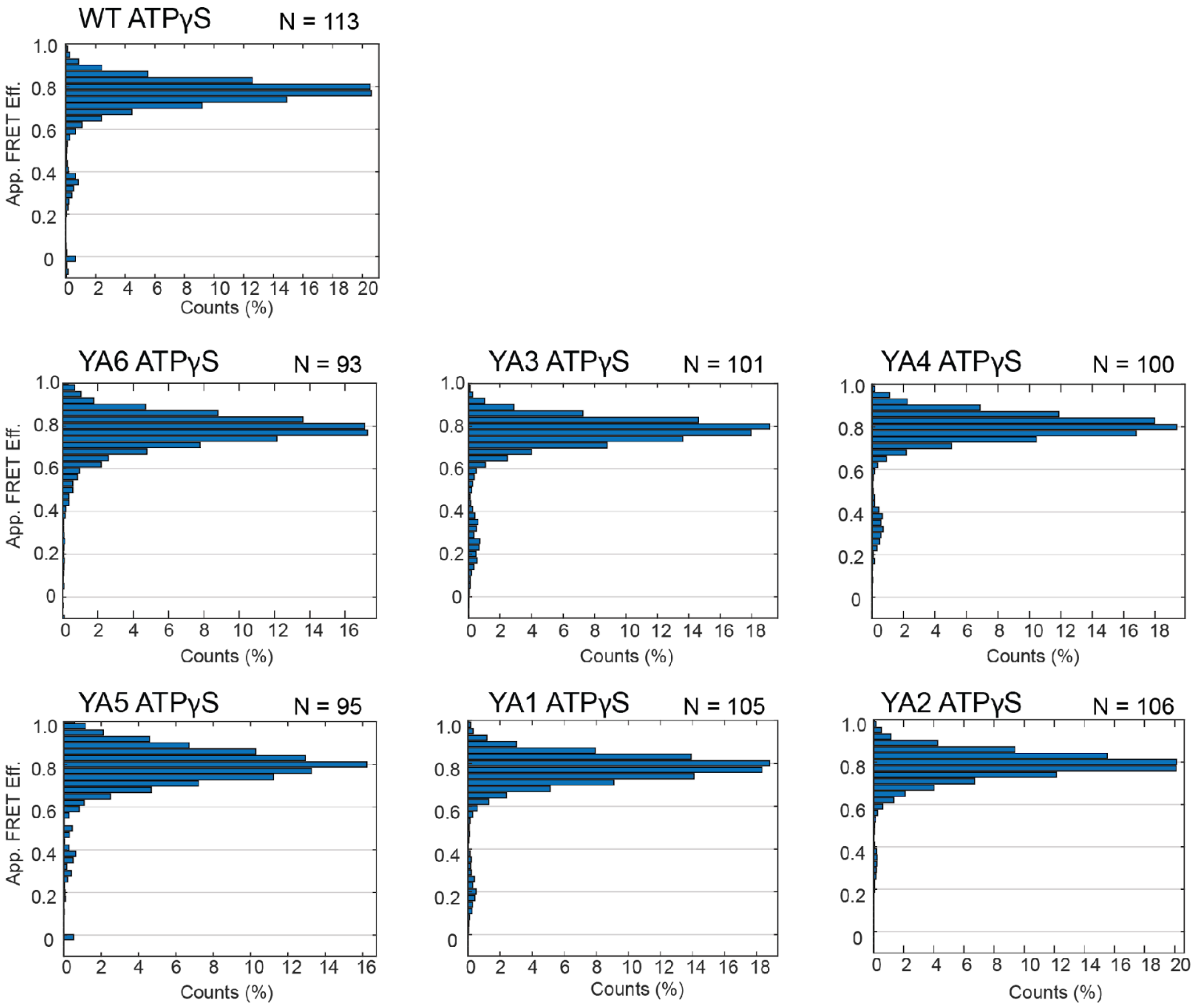

Supplementary Fig. 9. Apparent FRET efficiency distributions for wild-type and mutant proteasomes in the presence of the non-hydrolysable ATP analog ATPγS.

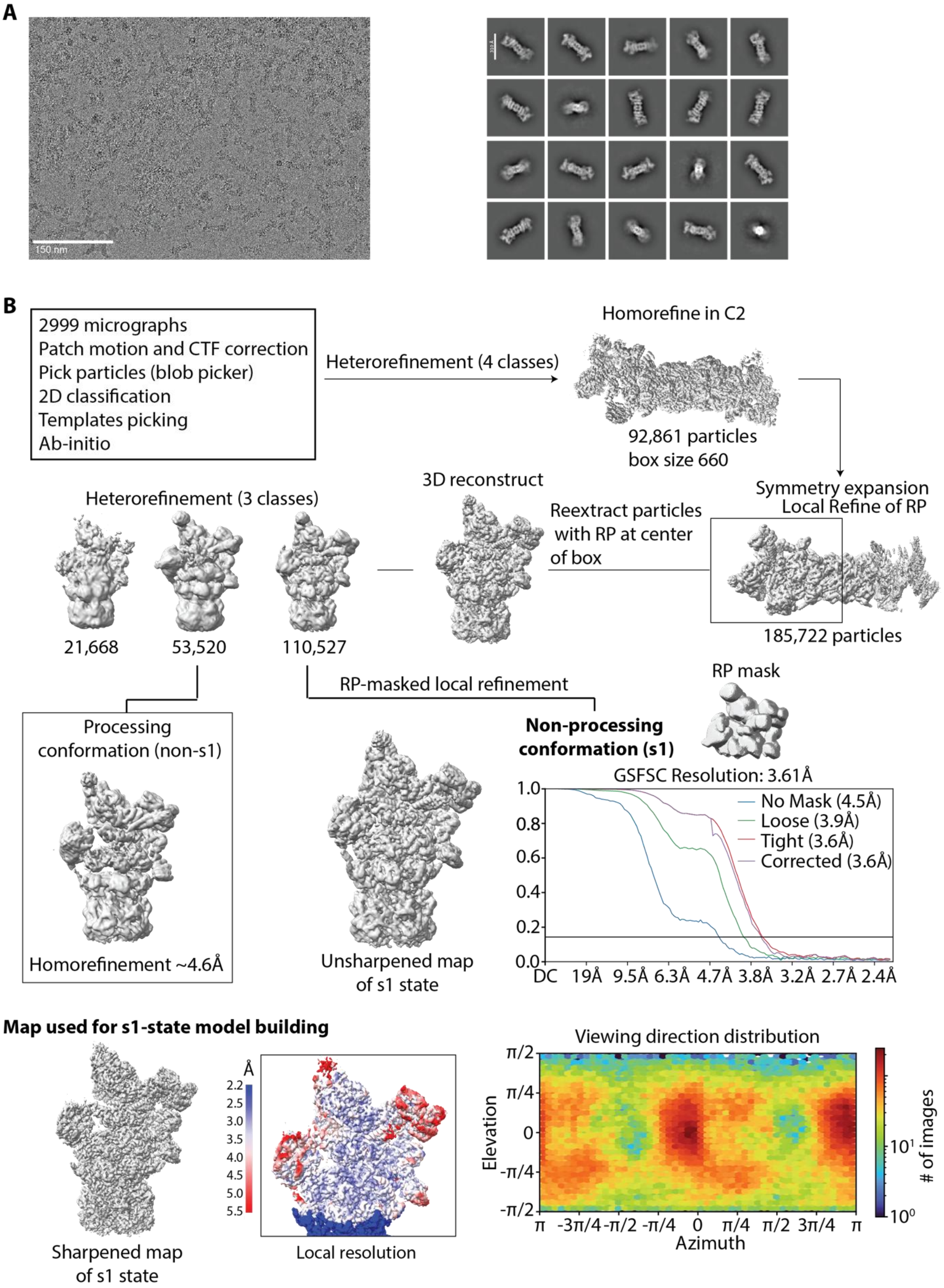
A
150 nm
B
2999 micrographs
Patch motion and CTF correction
Pick particles (blob picker)
2D classification
Templates picking
Ab-initio
Heterorefinement (4 classes)
Homorefine in C2
92,861 particles
box size 660
Symmetry expansion
Local Refine of RP
185,722 particles
Reextract particles
with RP at center
of box
3D reconstruct
Heterorefinement (3 classes)
21,668
53,520
110,527
RP-masked local refinement
RP mask
Processing
conformation (non-s1)
Homorefinement ~4.6Å
Unsharpened map
of s1 state
Non-processing
conformation (s1)
GSFSC Resolution: 3.61Å
No Mask (4.5Å)
Loose (3.9Å)
Tight (3.6Å)
Corrected (3.6Å)
1.0
0.8
0.6
0.4
0.2
0.0
DC 19Å 9.5Å 6.3Å 4.7Å 3.8Å 3.2Å 2.7Å 2.4Å
Map used for s1-state model building
Sharpened map
of s1 state
Å
2.2
2.5
3.0
3.5
4.0
4.5
5.0
5.5
Local resolution
Viewing direction distribution
π/2
π/4
0
-π/4
-π/2
Elevation
π -3π/4 -π/2 -π/4 0 π/4 π/2 3π/4 π
Azimuth
# of images
10^1
10^0

Supplementary Fig. 10. Processing workflow for the cryo-EM structure determination of the substrate-free yeast 26S proteasome in the s1 state. A) After collecting the dataset of native *S.c.* 26S proteasomes on a Talos Arctica 200kV, processing with cryoSPARC v4.4.0, and blob picking, 2D classification was used to generate templates for template picking. An example micrograph is shown on the left and 2D class averages on the right. **B)** Data processing workflow. Particles were extracted with a 660 box size and heterorefinement with input *ab initio* models was used to sort particles. Particles for 30S proteasomes were refined with C2 symmetry, and C2 symmetry expansion was used to effectively double the number of particles, followed by re-extraction with a 340 box size of the 19S RP recentered coordinates. 3D classification with heterorefinement generated three broad classes with either junk, non-s1, or s1-state particles. Non-s1-state particles were refined but not processed further due to limiting resolution. Particles belonging to the s1 conformation were locally refined with non-uniform refinement using an RP-focused mask. The sharpened map from cryoSPARC refinement was used for model building in Coot. The Gold-Standard FSC plot and distribution of particles are shown on the bottom right. All maps are displayed using ChimeraX.

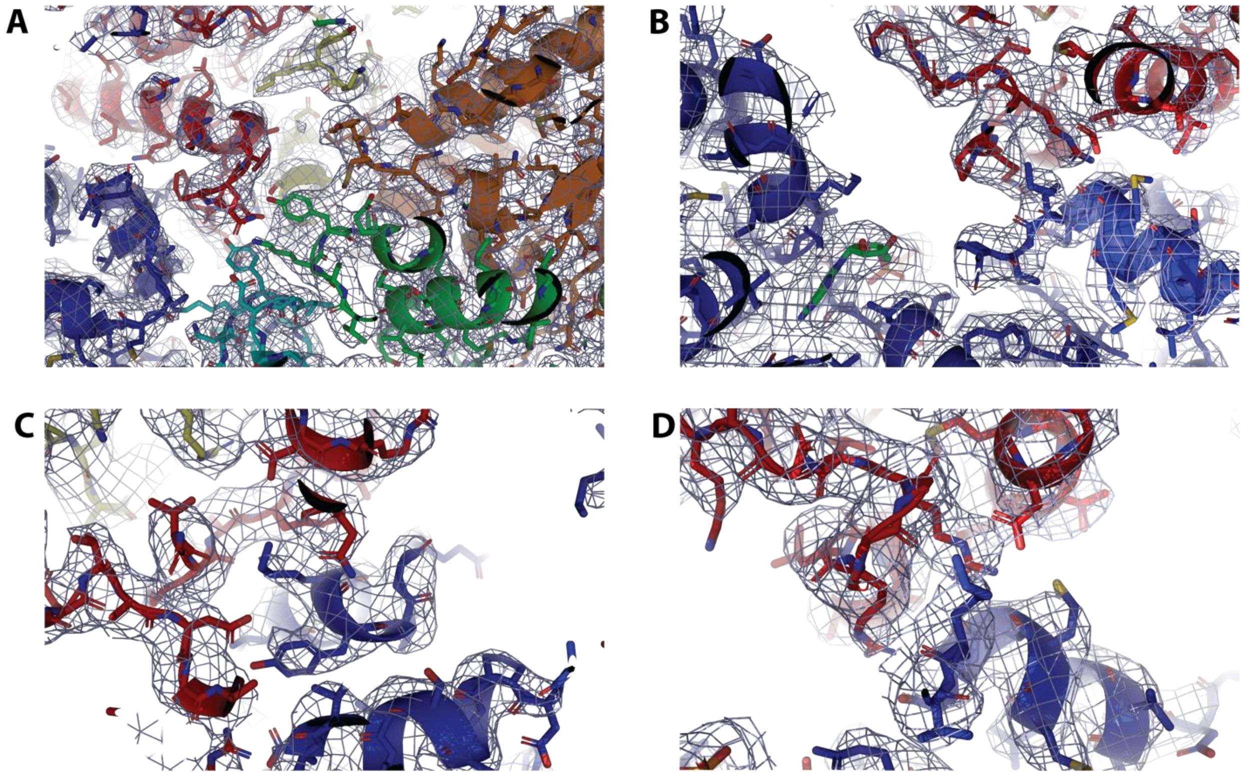

Supplementary Fig. 11. Cryo-EM map of the s1-state yeast 26S proteasome and docked atomic model for the ATPase hexamer. Shown are a focus on the central channel with Rpt1 in green, Rpt2 in cyan, Rpt6 in blue, Rpt3 in red, Rpt4 in yellow, and Rpt5 in orange (**A**), the Rpt6/Rpt3 interface (B), the helical conformation of Rpt6's pore-1 loop and its interaction with the second region of homology (SRH) of Rpt3 (**C**), and Rpt3's SRH with its Arg fingers contacting Rpt6 (**D**).

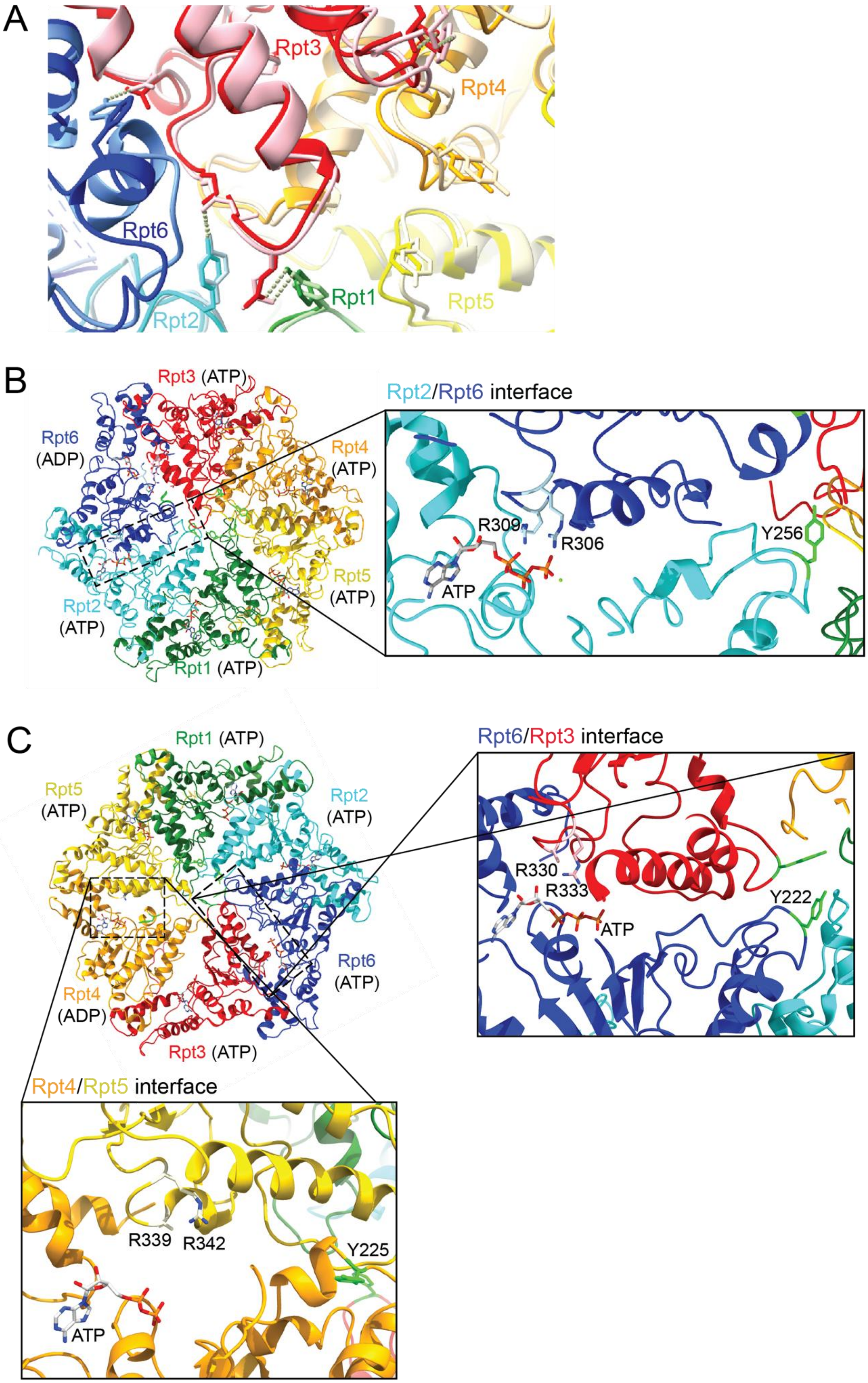
A
Rpt3
Rpt4
Rpt6
Rpt1
Rpt5
Rpt2
B
Rpt3 (ATP)
Rpt6
(ADP)
Rpt4
(ATP)
Rpt5
(ATP)
Rpt2
(ATP)
Rpt1 (ATP)
Rpt2/Rpt6 interface
R309
R306
Y256
ATP
C
Rpt1 (ATP)
Rpt5
(ATP)
Rpt2
(ATP)
Rpt6
(ATP)
Rpt4
(ADP)
Rpt3 (ATP)
Rpt6/Rpt3 interface
R330
R333
Y222
ATP
Rpt4/Rpt5 interface
R339
R342
Y225
ATP

Supplementary Fig. 12. Only the Rpt6/Rpt3 interface in the s1 state of the ATPase hexamer shows an arginine-finger mediated pore-1 loop stabilization. A) The helical conformation of Rpt6's pore-1 loop and hydrogen-bonds stabilizing the pore-1 loop tyrosines of Rpt1, Rpt2, and Rpt3 in the resting s1 state are conserved between yeast and human proteasomes. The structural model for the human 26S proteasome in the resting, engagement-competent state (dark color shadings, PDB ID: 8USB) is overlayed with the resting s1-state structure of the yeast 26S proteasome solved in this study (light shading, PDB ID: 9CGC). Due to the lack of the hydroxyl group, the pore-1 loop F223 of Rpt6 in the human proteasome is not hydrogen-bonded to an aspartate in Rpt3 like Y222 in the yeast Rpt6. **B)** Focus on the Rpt2/Rpt6 interface, in which the arginine fingers of Rpt6, R206 and R308, show the typical orientation towards the ATP nucleotide bound to the counterclockwise-neighboring Rpt2 subunit, far away from the pore-1 loop Y256 of Rpt2. **C)** Rpt-subunit interfaces in the substrate-engaged, non-s1-state proteasome (4D state, PDB ID: 6EF3) show no arginine-finger mediated pore-1 loop stabilization. **Top left:** The Rpt subunits are arranged in a spiral staircase with Rpt5 (yellow) at the top, Rpt3 (red) at the bottom, and Rpt4 (orange) in the seam position. All subunits are bound to ATP, except for the ADP-bound Rpt4. Nucleotides, the pore-1 loop tyrosines (green), and arginine fingers of Rpt5 and Rpt3 are shown in stick representation. **Bottom left:** Zoom-in on the seam-subunit interface between Rpt4 and Rpt5, which shows Rpt5's arginine fingers R339 and R342 in proximity to the Rpt4-bound ADP and thus far away from the Rpt4's pore-1 loop Y225. **Top right:** Zoom-in on the interface between Rpt6 and Rpt3, showing Rpt3's arginine fingers R330 and R333 in proximity to the ATP bound to Rpt6 and therefore at a large distance from Rpt6's pore-1 loop.

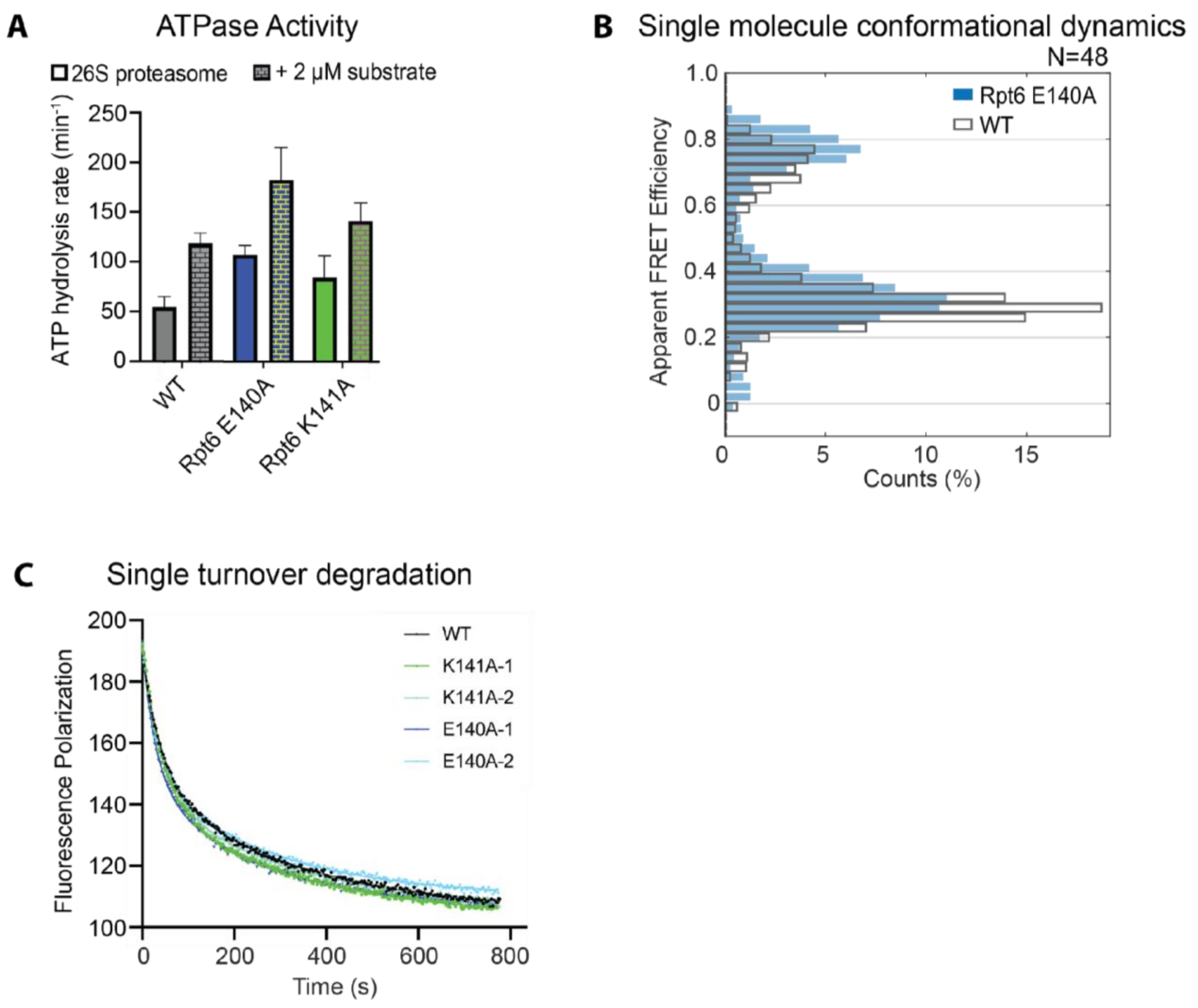

Supplementary Fig. 13. Rpt6 mutations at the interface to Rpt3 shift the proteasome conformational equilibrium and stimulate ATPase activity. A) Rates for ATPase activity of reconstituted wild-type proteasomes and proteasomes with E140A or K141A-mutant Rpt6 in the absence (solid bars) and presence of 2 μM ubiquitinated titin I27^{V15P} substrate (dashed bars). Technical replicates (n = 3) are plotted with error bars representing the SEM. **B)** Apparent FRET efficiency distributions for Rpt6 E140A mutant proteasomes in ATP (blue) compared to the distribution of the wild-type proteasome (grey outlined bars). **C)** Representative traces for the single-turnover degradation of the ubiquitinated FAM-Titin I27^{V15P} substrate by *in vitro*-reconstituted wild-type proteasomes and proteasomes with E140A or K141A mutation in Rpt6, monitored by the decrease in fluorescence polarization.

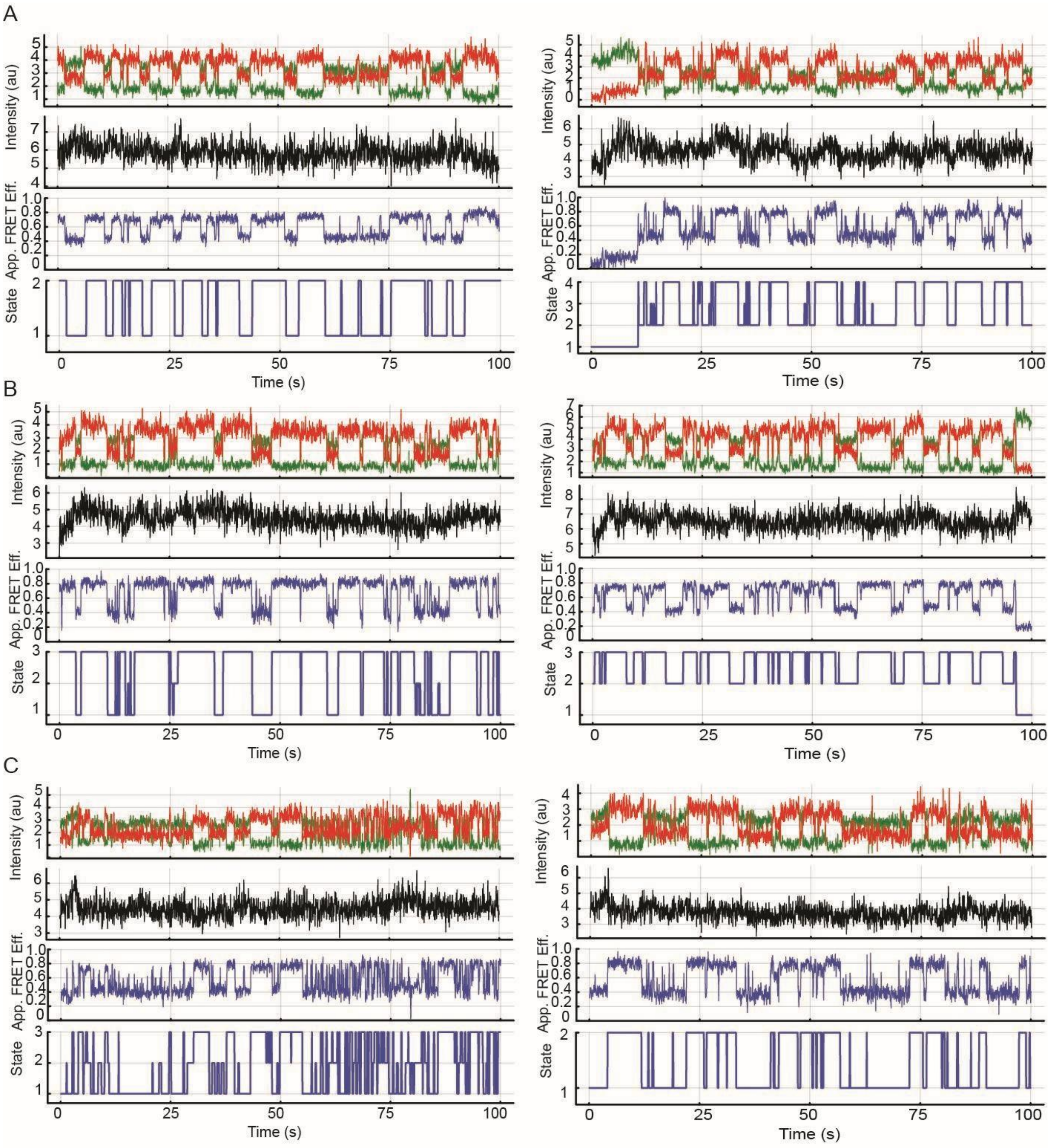

Supplementary Fig. 14. Representative traces from the conformational dynamics assay monitoring reconstituted wild-type **(A)**, YA6 **(B)**, and YA4-mutant proteasomes **(C)** in the presence of 1 μM titin I27^{V15P} substrate. The first panels show fluorescence intensities for the FRET donor (green) and acceptor (red), the second panels depict the total fluorescence intensities (black), the

third panels show the apparent FRET efficiencies, and the fourth panels illustrate the estimated most-probable state trajectories as determined by the BNP-FRET algorithm.

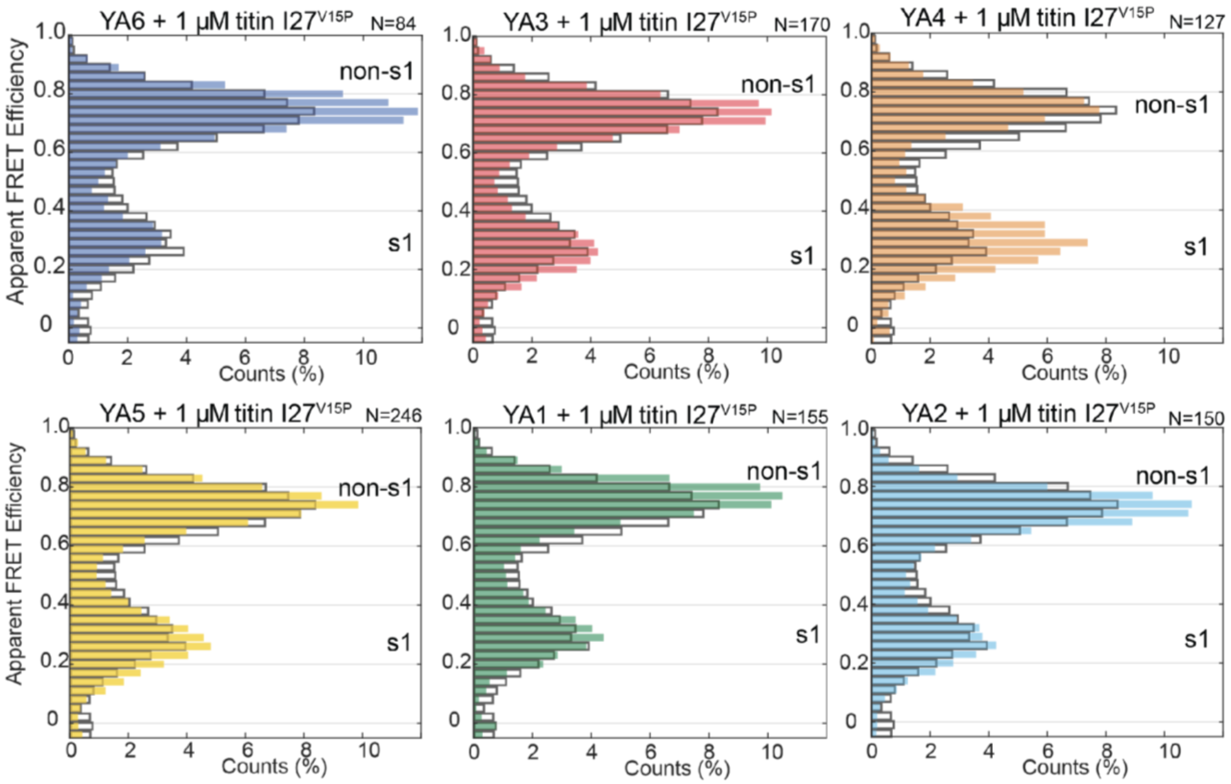

Supplementary Fig. 15. Apparent FRET efficiency distributions for YA6 (dark blue), YA3 (red), YA4 (orange), YA5 (yellow), YA1 (green), and YA2 (light blue) mutant proteasomes in the presence of 1 μM titin I27^{V15P} substrate compared to the distribution of the wild-type proteasome (grey outlined bars).

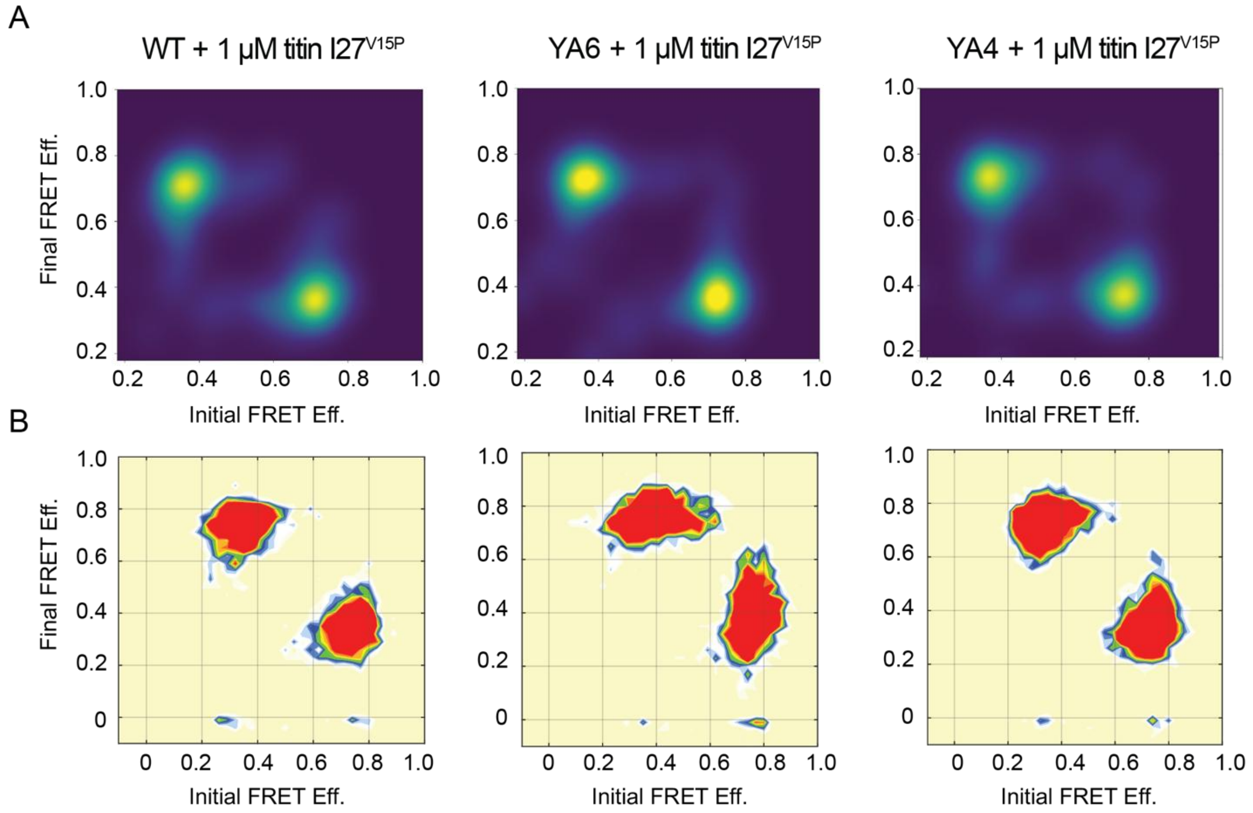

Supplementary Fig. 16. Transition density plots for wild-type, YA6, and YA4-muant 26S proteasomes in the presence of 1 μM titin I27^{V15P} substrate determined by the (A) BNP-FRET algorithm or (B) the Spartan software.

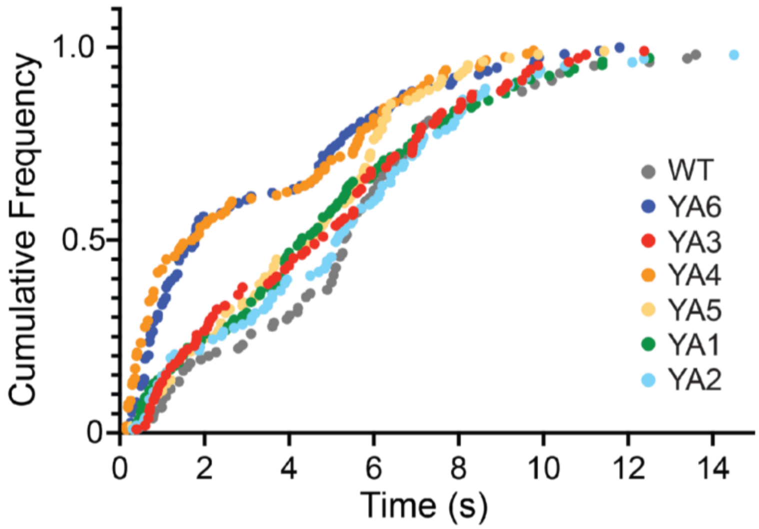

Supplementary Fig. 17. Cumulative frequencies for the dwell times of the high-FRET phases for WT and YA-mutant proteasomes in the presence of 0.5 μM titin I27^{V15P} substrate.

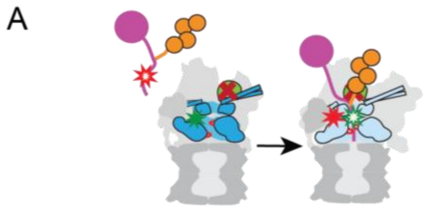

Variant	Tau (sec)	Cy3 Fluorescence Amplitude Change
WT	6.8 ± 0.1	0.095 ± 0.004
YA6	18.0 ± 2.5	0.019 ± 0.004
YA4	14.3 ± 0.8	0.077 ± 0.005

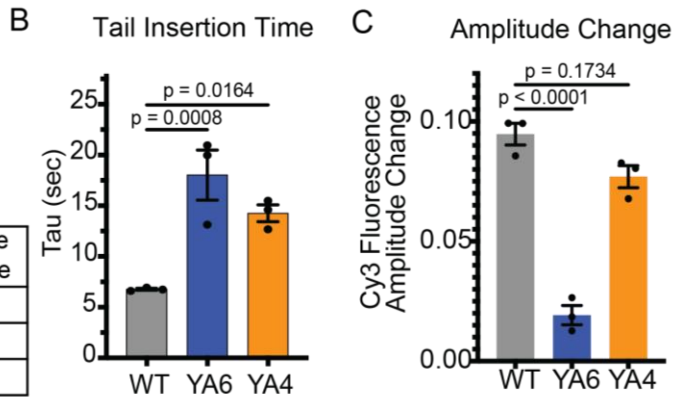

Supplementary Fig. 18. A) Top: Schematic for the ensemble measurements of substrate-tail insertion by monitoring the FRET efficiency between SulfoCy3-labeled 26S proteasomes with inhibited Rpn11 deubiquitinase (indicated by a red cross) and SulfoCy5-labeled substrate. The substrate and reconstituted proteasomes were rapidly mixed in a stopped flow instrument. Bottom: Time constants and Cy3-fluorescence amplitude changes (AU) during substrate tail insertion and engagement by wild-type and YA-mutant proteasomes. The error represents the SEM. **B)** Plotted time constants for tail insertion and **C)** Cy3-fluorescence amplitude changes obtained from the single exponential fitting of curves shown in Fig. 5B ($n \geq 3$, technical replicates, error bars represent the SEM, statistical significance was calculated using a one-way ANOVA test with the p values indicated).

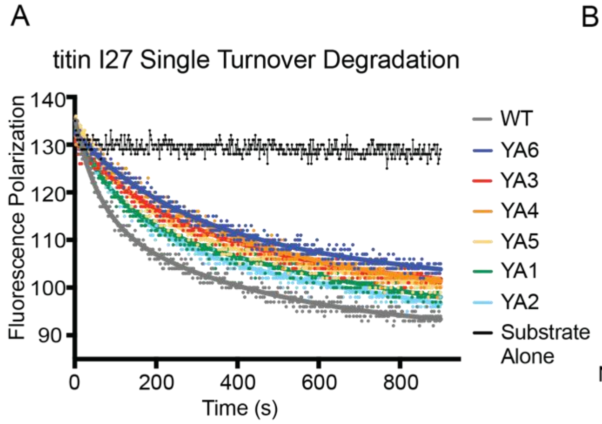

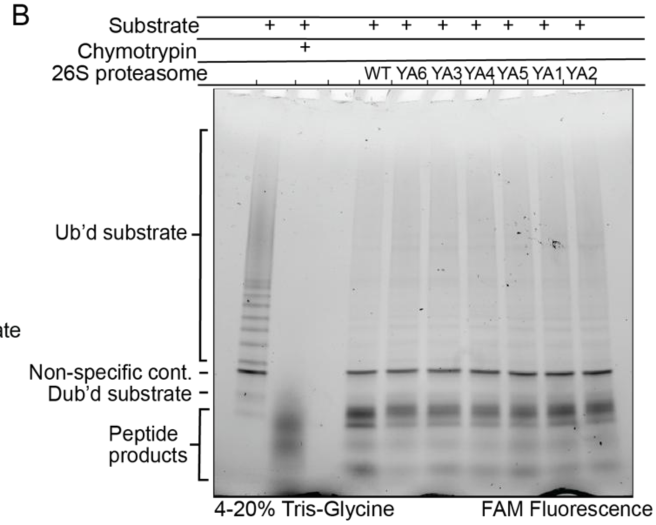

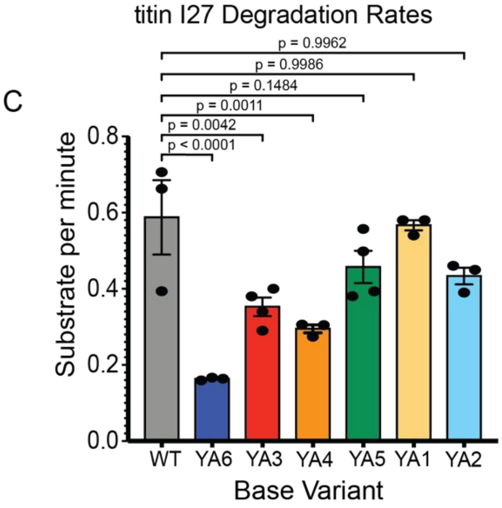

Variant	titin I27 Single Turnover Degradation Rate (substrate enzyme^{-1} min^{-1})
WT	0.62 ± 0.10
YA6	0.16 ± 0.01
YA3	0.35 ± 0.04
YA4	0.30 ± 0.05
YA5	0.43 ± 0.04
YA1	0.57 ± 0.09
YA2	0.47 ± 0.07

Supplementary Fig. 19. A) Representative traces for the single-turnover degradation of ubiquitinated FAM-titin I27 substrate by *in vitro*-reconstituted proteasomes. **B)** SDS-PAGE visualization of end-point samples from the single-turnover degradation of the FAM-titin I27 substrate by *in vitro*-reconstituted proteasomes. **C)** Single-turnover degradation rates obtained from fitting the curves shown in panel (A) to a double exponential decay using GraphPad Prism. (n = 3, technical replicates, error bars represent the SEM, statistical significance was calculated using a one-way ANOVA test with the p values indicated).

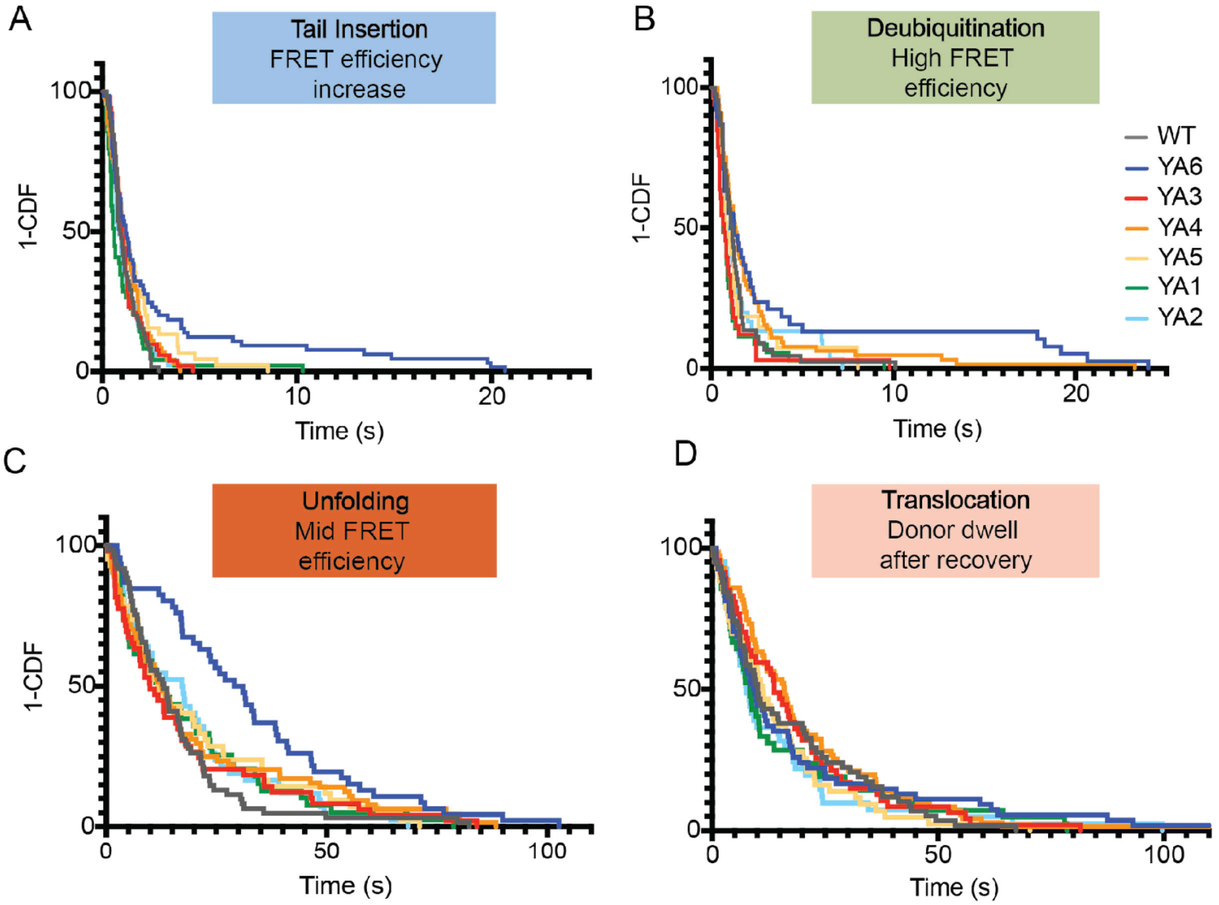

Supplementary Fig. 20. Single-molecule FRET-based measurements of titin I27 substrate processing by wild-type and YA-mutant proteasomes. 1-CDF (1-cumulative distribution function) or survival plot analyses for **A)** substrate tail insertion, **B)** deubiquitination, **C)** unfolding, and **D)** translocation after unfolding.

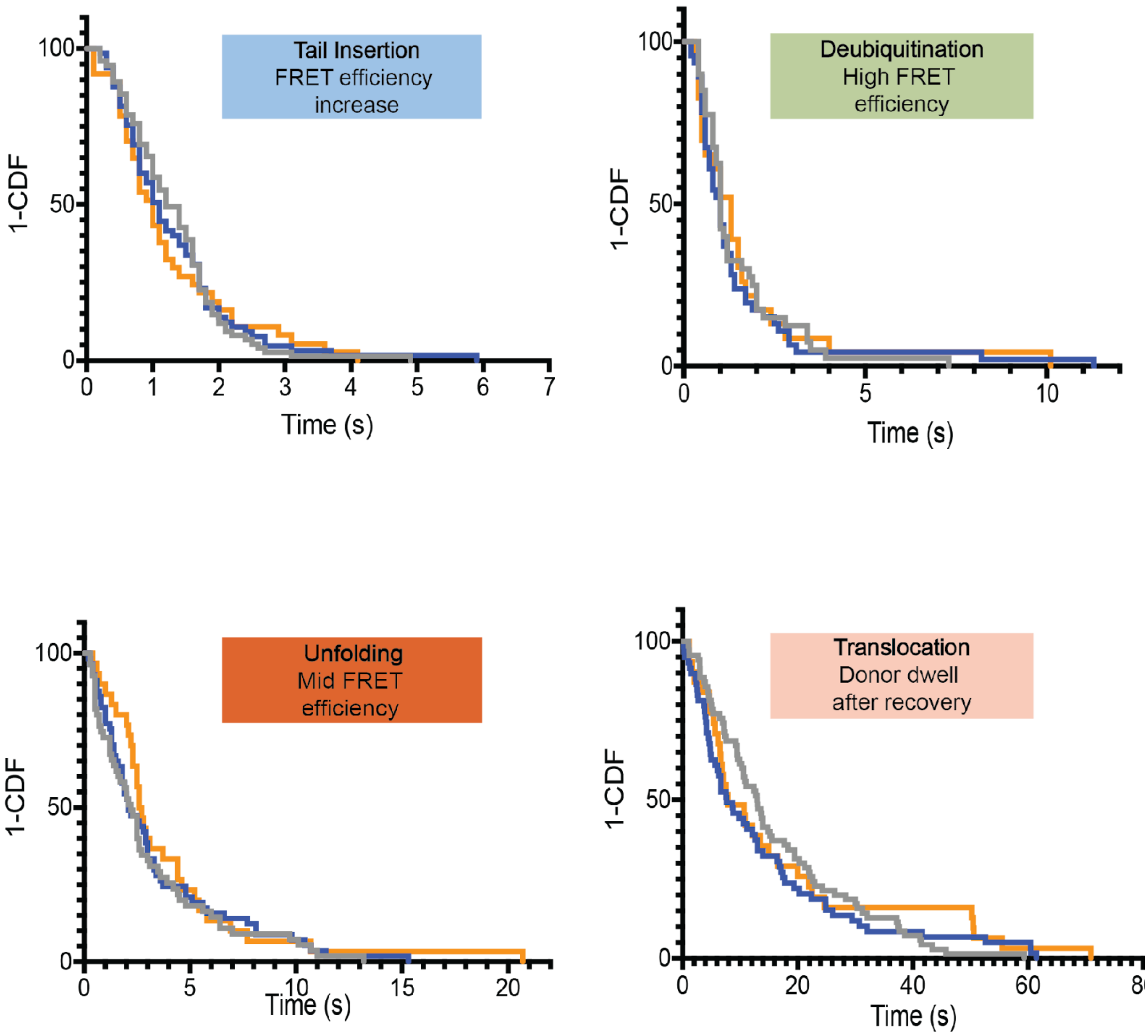

Supplementary Fig. 21. Single-molecule FRET-based measurements of titin I27^{V15P} substrate processing by wild-type and YA-mutant proteasomes. 1-CDF (1-cumulative distribution function) or survival plot analyses for **A)** substrate tail insertion, **B)** deubiquitination, **C)** unfolding, and **D)** translocation after unfolding.

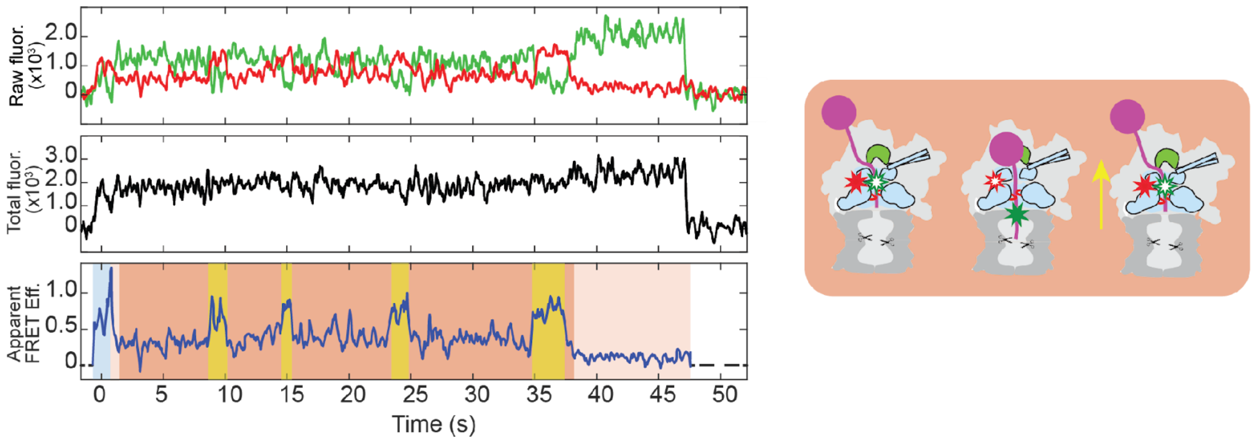

Supplementary Fig. 22. Backsliding events (yellow shading) of the titin I27 substrate during the unfolding phase (orange shading) by the YA3-mutant proteasomes, observed by single-molecule FRET.

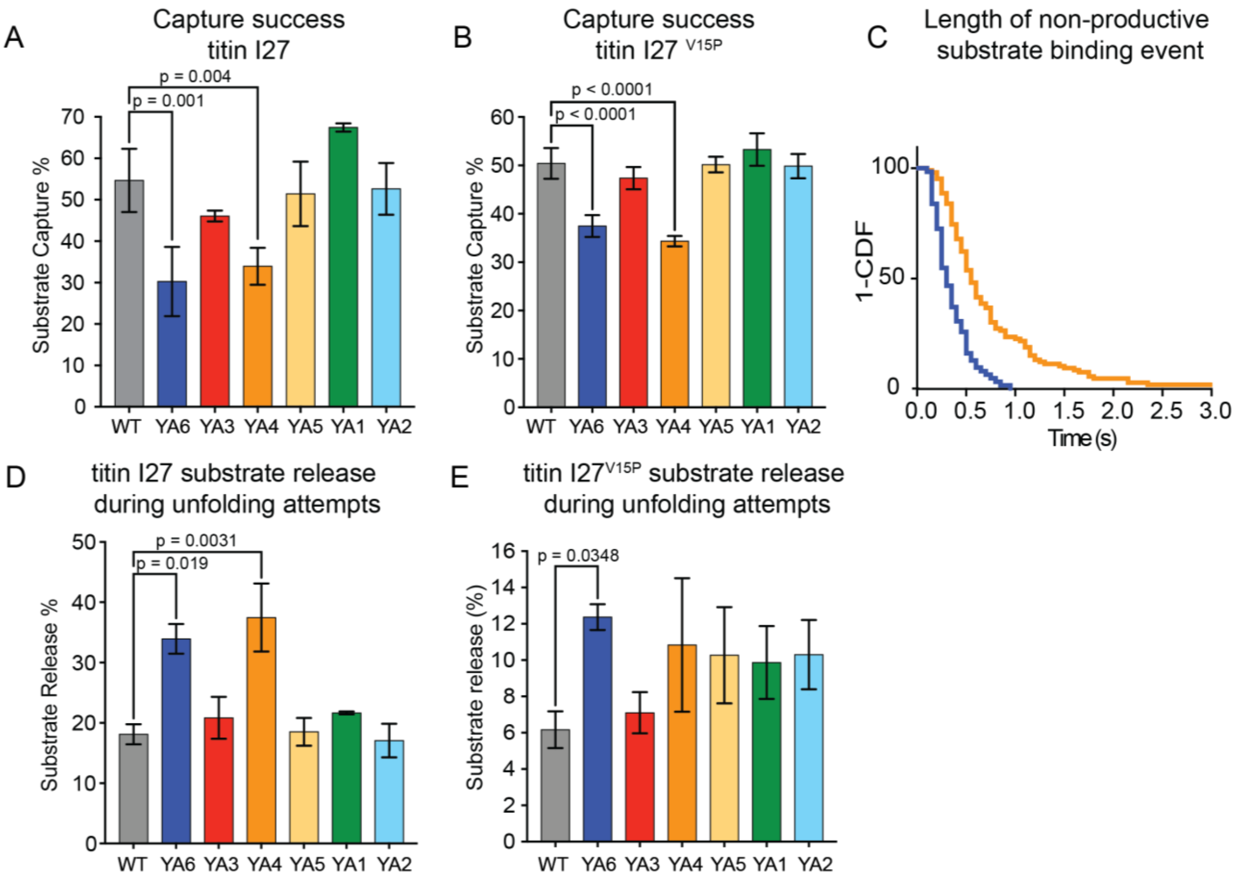

Supplementary Fig. 23. Success rates for the capture of wild-type titin I27 **(A)** and titin I27^{V15P} substrate **(B)** by wild-type and YA-mutant proteasomes. For these calculations, n = 3 technical replicates were obtained, contributing to N ≥ 210 events for wild-type titin I27 and N > 300 for titin I27^{V15P}. Error bars represent the SD, and statistical significance was calculated using a one-way ANOVA test. **C)** Survival plots for brief events of substrate binding to YA4 and YA6-mutant proteasomes that do not lead to successful degradation. (N = 106 for YA4, N = 62 for YA6) **D)** Percent of wild-type titin I27 substrate release during unfolding attempts by wild-type and YA-mutant proteasomes. n = 3 technical replicates were performed, contributing to N > 105 events. Statistical significance was calculated using a one-way ANOVA test. **E)** Percent of titin I27^{V15P} substrate release during unfolding attempts by wild-type and pore-loop mutant

proteasomes. n = 3 technical replicates were measured, contributing to N $\geq$ 210 events.

Statistical significance was calculated using a one-way ANOVA test.

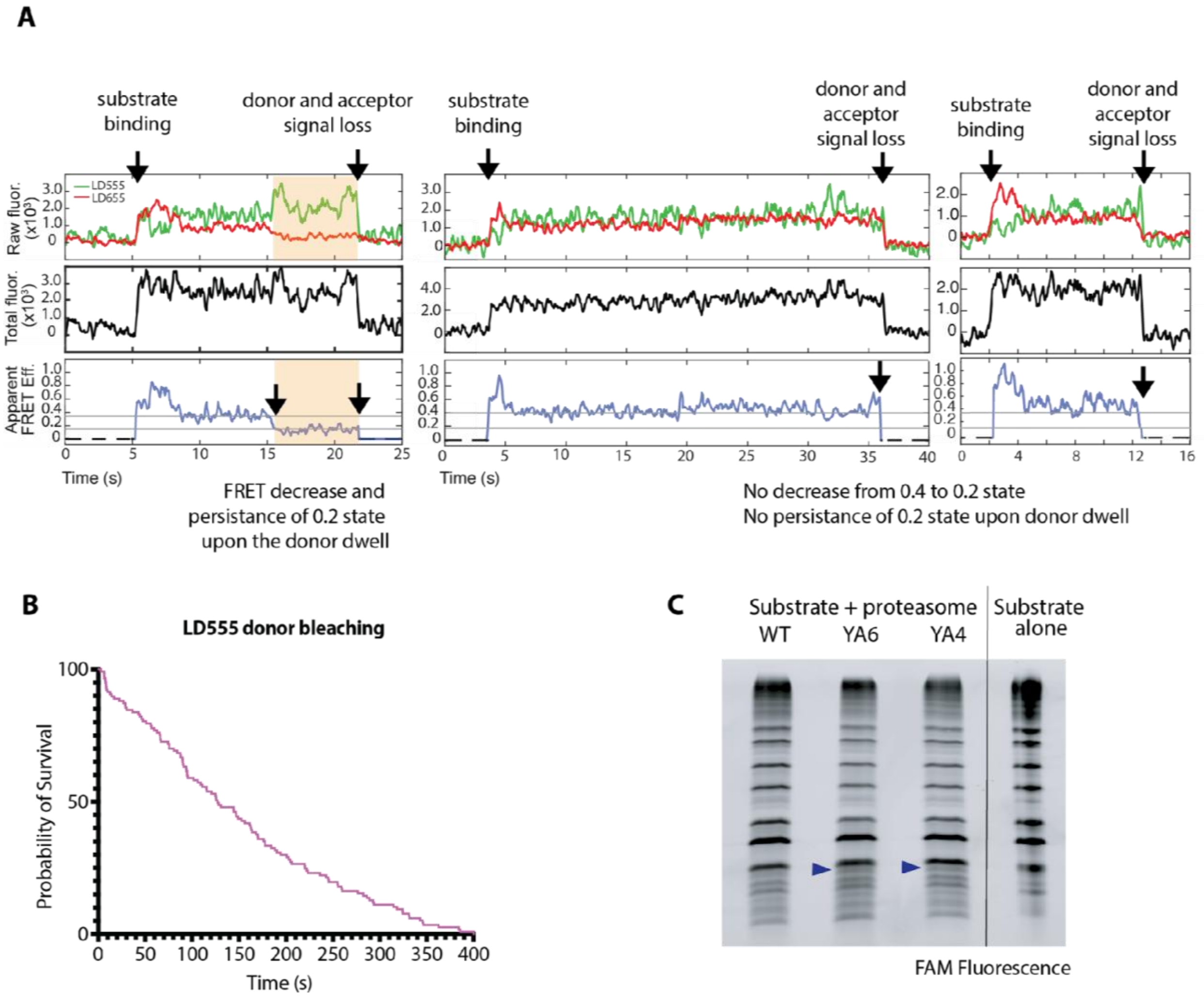

Supplementary Fig. 24. Substrate-release calculations. **A)** Substrate degradation events were scored as previously described. Traces on the left show a successful degradation event, with the FRET efficiency decreasing from ~ 0.40 to ~ 0.20 (signifying a progression from the pre-unfolding dwell to translocation of the unfolded protein) and then persisting at ~ 0.2 during translocation. Substrate release (middle and right traces) was evaluated as the abrupt loss of donor and acceptor signal during the unfolding dwell, i.e. at the ~ 0.40 FRET-efficiency state, with no decrease in FRET signal from ~ 0.40 to ~ 0.20 or a persistent 0.2 FRET efficiency. **B)** Measurement of the LD555 donor life time using LD555-labeled proteasomes in the absence of acceptor. A total of 152 particles were analyzed, out of which 26 % (N = 39) did not show donor photobleaching within the monitored time of 400 s. The remaining 78 % (N = 113) of

photobleaching events were plotted to calculate a $t_{1/2}$ ~ 127 s. **C)** SDS-PAGE analysis of the 60-min end point samples from the multiple-turnover degradation of ubiquitinated FAM-labeled titin I27 substrate by wild-type, YA6, and YA4-mutant proteasomes. The accumulation of deubiquitinated and truncated products (indicated by arrowheads) was visualized by FAM fluorescence.

Supplementary tables:

WT (ATP)	Mean λesc (s^{-1})	S.D. (s^{-1})	Population weight	Coefficient of variation
s1 escape rates	4.22	1.39	0.036	0.33
	1.91	0.68	0.266	0.36
	0.81	0.30	0.226	0.37
	0.34	0.13	0.233	0.40
non-s1 escape rates	7.22	1.34	0.014	0.19
	2.90	0.90	0.340	0.31
	1.80	0.61	0.409	0.34
	0.66	0.28	0.199	0.42

Supplementary Table 1. Escape rates of proteasome conformational switching determined by BNP-FRET for WT 26S proteasomes in the absence of substrate (2 mM ATP). Traces resulting from the FRET-based conformational change assay for wild-type proteasomes were analyzed using BNP-FRET techniques to determine the escape rates (λesc (s^{-1})) for the conformational transitions from the s1 to non-s1 states and from non-s1 to s1 states.

YA4 (ATP)	Mean λesc (s^{-1})	S.D. (s^{-1})	Population weight	Coefficient of variation
s1 escape rates	4.71	1.44	0.033	0.30
	2.60	0.51	0.096	0.20
	1.07	0.38	0.238	0.35
	0.36	0.19	0.296	0.52
non-s1 escape rates	5.11	1.58	0.053	0.31
	2.84	1.01	0.342	0.35
	1.27	0.53	0.342	0.41
	0.42	0.20	0.202	0.47

Supplementary Table 2. Escape rates of proteasome conformational switching determined by BNP-FRET for YA4 mutant 26S proteasomes in the absence of substrate (2 mM ATP). Traces resulting from the FRET-based conformational change assay for YA4 mutant proteasomes were analyzed using BNP-FRET techniques to determine the escape rates (λesc (s^{-1}))) for the conformational transitions from the s1 to non-s1 states and from non-s1 to s1 states.

YA6 (ATP)	Mean λesc (s^{-1})	S.D. (s^{-1})	Population weight	Coefficient of variation
s1 escape rates	4.90	1.75	0.063	0.36
	2.69	0.92	0.139	0.34
	1.21	0.43	0.221	0.36
	0.47	0.17	0.310	0.37
non-s1 escape rates	5.43	1.87	0.061	0.34
	3.29	0.65	0.127	0.20
	1.75	0.56	0.541	0.32
	0.69	0.32	0.208	0.46

Supplementary Table 3. Escape rates of proteasome conformational switching determined by BNP-FRET for YA6 mutant 26S proteasomes in the absence of substrate (2 mM ATP). Traces resulting from the FRET-based conformational change assay for YA6 mutant proteasomes were analyzed using BNP-FRET techniques to determine the escape rates (λesc (s^{-1}))) for the conformational transitions from the s1 to non-s1 states and from non-s1 to s1 states.

Proteasome variant	s1 escape rates (mean + S.D.)		non-s1 escape rates (mean + S.D.)	
	k_{fast} (s^{-1})	k_{slow} (s^{-1})	k_{fast} (s^{-1})	k_{slow} (s^{-1})
WT + 2mM ATP	2.55 ± 0.41	0.43 ± 0.03	9.57 ± 1.39	2.12 ± 0.23
YA6 + 2mM ATP	3.80 ± 0.46	0.62 ± 0.12	6.66 ± 0.82	1.54 ± 0.19
YA4 + 2mM ATP	2.83 ± 0.14	0.51 ± 0.09	8.47 ± 0.48	1.94 ± 0.12

Supplementary Table 4. Escape rates of proteasome conformational switching determined by ebFRET. Traces resulting from the FRET-based conformational change assay for wild-type and pore-1 loop mutant proteasomes in the absence (ATP) of ubiquitinated titin I27^{V15P} were analyzed using ebFRET to determine the rates (k) for the conformational transitions from the s1 to non-s1 states (s1 escape rates) and from non-s1 to s1 states (non-s1 escape rates).

WT (1 μM Titin I27^{V15P})	Mean λesc (s^{-1})	S.D. (s^{-1})	Population weight	Coefficient of variation
s1 escape rates	6.12	1.97	0.006	0.32
	4.73	1.17	0.099	0.25
	2.66	0.78	0.098	0.29
	1.48	0.48	0.288	0.33
	0.66	0.24	0.262	0.36
non-s1 escape rates	6.61	1.03	0.015	0.16
	3.18	1.28	0.028	0.40
	1.11	0.42	0.161	0.38
	0.55	0.20	0.318	0.36

Supplementary Table 5. Escape rates of proteasome conformational switching determined by BNP-FRET for WT 26S proteasomes in the presence of 1μM Titin I27^{V15P} substrate.

YA4 (1 μM Titin I27^{V15P})	Mean λesc (s^{-1})	S.D. (s^{-1})	Population weight	Coefficient of variation
s1 escape rates	8.05	2.23	0.022	0.28
	3.34	1.42	0.115	0.43
	1.10	0.42	0.275	0.38
	0.55	0.21	0.234	0.39
non-s1 escape rates	10.90	1.84	0.007	0.17
	4.32	1.10	0.038	0.25
	2.26	0.73	0.177	0.32
	1.10	0.36	0.189	0.33
	0.64	0.23	0.375	0.36

Supplementary Table 6. Escape rates of proteasome conformational switching determined by BNP-FRET for YA4 mutant 26S proteasomes in the presence of 1 μM Titin I27^{V15P} substrate.

YA6 (1μM Titin I27^{V15P})	Mean λesc (s^{-1})	S.D. (s^{-1})	Population weight	Coefficient of variation
s1 escape rates	7.19	2.11	0.031	0.29
	3.32	1.07	0.153	0.32
	1.62	0.58	0.330	0.36
	0.78	0.33	0.433	0.42
non-s1 escape rates	8.72	1.13	0.012	0.13
	7.36	0.71	0.003	0.10
	4.46	1.28	0.034	0.29
	1.42	0.59	0.091	0.41
	0.38	0.22	0.861	0.57

Supplementary Table 7. Escape rates of proteasome conformational switching determined by BNP-FRET for YA6 mutant 26S proteasomes in the presence of 1μM Titin I27^{V15P} substrate.

**26S proteasome variant**	+0.5 μM Ub'ed titin I27^{V15P} (mean ± SEM)	+0.5 μM Ub'ed titin I27^{V15P} N	+1.0 μM ub'd titin I27^{V15P} (mean ± SEM)	+1.0 μM Ub'ed titin I27^{V15P} N
WT	5.48 ± 0.32	105	7.21 ± 0.45	74
YA6	3.18 ± 0.28	114	6.54 ± 0.52	84
YA3	4.83 ± 0.33	106	7.99 ± 0.47	78
YA4	3.08 ± 0.28	120	6.73 ± 0.52	90
YA5	4.40 ± 0.27	109	7.15 ± 0.38	79
YA1	4.96 ± 0.35	109	7.49 ± 0.45	81
YA2	5.21 ± 0.33	103	6.70 ± 0.30	90

Supplementary Table 8. Substrate processing dwells. Listed are the mean values for the duration of high-FRET processing dwells for degradation of titin I27^{V15P} at 0.5 μM and 1.0 μM measured by the FRET-based conformational change assay. The N values indicate the number of analyzed excursions to the non-s1 state and errors represent the SEM.

**26S proteasome variant**	**Tau (s)**	**95% CI**	**N**
WT	1.48	1.20 - 1.90	65
YA6	1.42	1.29 - 1.55	66
YA3	1.22	1.00 - 1.52	52
YA4	1.63	1.47 - 1.83	72
YA5	1.47	1.34 - 1.61	45
YA1	0.79	0.69 - 0.91	50
YA2	1.06	0.92 - 1.23	47

Supplementary Table 9. Titin I27 tail insertion engagement kinetics. Listed are the time constants for tail insertion and engagement of ubiquitinated titin I27 substrate, as determined by single exponential decay fitting of the FRET-based tail insertion phase length in the substrate processing assay. N values indicate the number of analyzed events.

26S proteasome variant	Tau (s)	95% CI	N
WT	1.29	1.09 - 1.54	44
YA6	1.49	1.37 - 1.63	39
YA3	0.85	0.73 - 0.98	33
YA4	1.51	1.39 - 1.63	64
YA5	1.03	0.84 - 1.29	27
YA1	0.81	0.65 - 1.03	36
YA2	1.10	0.86 - 1.19	30

Supplementary Table 10. Titin I27 deubiquitination kinetics. Listed are the time constants for deubiquitination of ubiquitinated titin I27 substrate, as determined by single exponential decay fitting of the FRET-based deubiquitination phase length in the substrate processing assay. N values indicate the number of analyzed events.

26S proteasome variant	Tau (s)	95% CI	N
WT	16.31	14.96 - 17.82	61
YA6	50.82	43.50 - 60.58	46
YA3	13.46	12.51 - 14.49	52
YA4	15.43	14.53 - 16.40	64
YA5	18.10	16.56 - 19.85	45
YA1	17.29	15.21 - 19.73	47
YA2	21.06	19.24 - 23.14	47

Supplementary Table 11. Titin I27 pre-unfolding kinetics. Listed are the time constants for pre-unfolding dwell of ubiquitinated titin I27 substrate, as determined by single exponential decay fitting of the FRET-based pre-unfolding phase length in the substrate processing assay. N values indicate the number of analyzed events.

26S proteasome variant	Tau (s)	95% CI	N
WT	15.27	13.85 - 16.91	58
YA6	11.73	11.09 - 12.42	54
YA3	19.04	17.91 - 20.26	47
YA4	20.88	19.61 - 22.28	71
YA5	16.16	15.02 - 17.41	43
YA1	10.13	9.26 - 11.13	43
YA2	10.84	9.43 - 12.53	41

Supplementary Table 12. Titin I27 translocation kinetics. Listed are the time constant values for translocation of ubiquitinated titin I27 substrate, as determined by single exponential decay fitting of the FRET-based translocation phase length in the substrate processing assay. N values indicate the number of analyzed events.

	Tail Insertion		Deubiquitination		Pre-unfolding		Translocation	
**26 proteasome variant**	**Tau (s)**	**N**	**Tau (s)**	**N**	**Tau (s)**	**N**	**Tau (s)**	**N**
WT	1.88 (1.45 - 2.58)	69	1.49 (1.13 - 2.03)	32	2.76 (2.57 - 2.97)	60	18.46 (17.27 - 19.80)	70
YA6	1.50 (1.25 - 1.81)	72	1.20 (1.00 - 1.45)	35	2.77 (2.58 - 2.98)	61	11.71 (11.05 - 12.42)	71
YA4	1.37 (1.09 - 1.79)	67	1.41 (1.09 - 1.85)	34	3.73 (3.06 - 4.61)	62	11.91 (10.48 - 13.61)	51

Supplementary Table 13. Titin I27^{V15P} substrate processing kinetics. Listed are the time constant values for tail insertion, deubiquitination, pre-unfolding dwell, and translocation of the ubiquitinated titin I27^{V15P} substrate, as determined by single exponential decay fitting of the FRET-based tail insertion phase length in the substrate processing assay. In parenthesis are the values for the 95% confidence interval. N values indicate the number of analyzed events.

26S proteasome variant	titin I27 capture success (%) + S.D.	titin I27 capture quantification (N)	titin I27^{V15P} capture success (%) + S.D.	titin I27^{V15P} capture quantification (N)
WT	54.7 ± 7.6	332	50.6 ± 3.4	348
YA6	30.2 ± 8.3	411	37.5 ± 2.3	314
YA3	46.1 ± 1.3	322	47.2 ± 2.4	306
YA4	33.9 ± 4.5	273	34.3 ± 1.0	302
YA5	51.4 ± 7.8	263	50.2 ± 1.6	302
YA1	67.4 ±1.0	210	53.2 ± 3.5	335
YA2	52.6 ± 6.2	283	49.7 ± 2.6	335

Supplementary Table 14. Substrate capture success by 26S proteasome variants. Listed are the average values and standard deviation for substrate capture success percentage of the ubiquitinated titin I27 and titin I27^{V15P} substrates. (N – number of quantified events; S.D. – standard deviation)

26S proteasome variant	titin I27 release (%) + S.D.	titin I27 release quantification (N)	titin $I27^{V15P}$ release (%) + S.D.	titin $I27^{V15P}$ release quantification (N)
WT	18.1 ± 2.9	145	6.2 ± 1.0	251
YA6	33.3 ± 5.1	160	12.4 ± 0.7	249
YA3	20.8 ± 6.0	169	7.1 ± 1.1	339
YA4	37.5 ± 9.8	132	10.8 ± 3.7	233
YA5	18.4 ± 3.9	122	9.9 ± 2.0	315
YA1	21.7 ± 0.4	106	10.3 ± 1.9	235
YA2	17.1 ± 4.8	140	10.3 ± 2.7	210

Supplementary Table 15. Substrate release upon pre-unfolding dwell 26S proteasome variants. Listed are the average values and standard deviation for substrate release percentage of the ubiquitinated titin I27 and titin $I27^{V15P}$ substrates. (N – number of quantified events; S.D. – standard deviation)

Study	Sample	Reported # of particles with Rpt4 pore-1 loop at the seam or bottom position (state according to author nomenclature)
De la Peña et al., 2018 [31]	*S. cerevisiae* 26S<br>+ Ub'ed titin $I27^{V15P}$<br>+ DUB inhibitor<br>+ ATP	30% (4D)<br>29% (5T)<br>19.2% (5D)
Dong et al., 2018 [45]	Human 26S<br>+ polyUb'ed-Sic1PY<br>+ ATP/ ATPγS	60% (E_{D2})<br>40% (E_{D1})
Zhang et al., 2022[37]	Human 26S<br>+ polyUb'ed-Sic1PY<br>+ Usp14<br>+ ATP/ATPγS	64% (E_{D2} $E_{D2.1}$)<br>12% (E_{D1})

Supplementary Table 16. Rpt4 pore-1 loop at the seam or bottom subunit of the AAA+ motor spiral staircase observed by cryo-EM studies. The percentage calculated is relative to the total number of particles observed in processing-states. For these studies. Yeast or human 26S proteasome were incubated with a substrate and stalled using different methods, either the deubiquitinase inhibitor 1,10-phenanthroline or mixture of ATP and the non-hydrolysable ATP analog ATPγS. In parenthesis on th4e third column is the nomenclature assigned by the authors for each processing state.

Plasmid ID	Phenotype	Source
pAM371	pCOLADuet-1 FLAG-Rpt1-Y283A, Rpt2, His_6-Rpt3, Rpt5, Rpt6, Rpt4	This study
pAM372	pCOLADuet-1 FLAG-Rpt1, Rpt2-Y256A, His_6-Rpt3, Rpt5, Rpt6, Rpt4	This study
pAM373	pCOLADuet-1 FLAG-Rpt1, Rpt2, His_6-Rpt3-Y246A, Rpt5, Rpt6, Rpt4	This study
pAM374	pCOLADuet-1 FLAG-Rpt1, Rpt2, His_6-Rpt3, Rpt5, Rpt6, Rpt4-Y256A	This study
pAM375	pCOLADuet-1 FLAG-Rpt1, Rpt2, His_6-Rpt3, Rpt5-Y255A, Rpt6, Rpt4	This study
pAM376	pCOLADuet-1 FLAG-Rpt1, Rpt2, His_6-Rpt3, Rpt5, Rpt6-Y222A, Rpt4	This study
pAM82	pCOLADuet-1 FLAG-Rpt1, Rpt2, His_6-Rpt3, Rpt5, Rpt6, Rpt4	[17]
pAM377	pCOLADuet-1 FLAG-Rpt1-I191TAG-Y283A, Rpt2, His_6-Rpt3, Rpt5, Rpt6, Rpt4	This study
pAM378	pCOLADuet-1 FLAG-Rpt-I191TAG, Rpt2-Y256A, His_6-Rpt3, Rpt5, Rpt6, Rpt4	This study
pAM379	pCOLADuet-1 FLAG-Rpt-I191TAG, Rpt2, His_6-Rpt3-Y246A, Rpt5, Rpt6, Rpt4	This study
pAM380	pCOLADuet-1 FLAG-Rpt1-I191TAG, Rpt2, His_6-Rpt3, Rpt5, Rpt6, Rpt4-Y256A	This study
pAM381	pCOLADuet-1 FLAG-Rpt1-I191TAG, Rpt2, His_6-Rpt3, Rpt5-Y255A, Rpt6, Rpt4	This study
pAM382	pCOLADuet-1 FLAG-Rpt1-I191TAG, Rpt2, His_6-Rpt3, Rpt5, Rpt6-Y222A, Rpt4	This study
pAM88	pCOLADuet-1 FLAG-Rpt1-I191TAG, Rpt2, His_6-Rpt3, Rpt5, Rpt6, Rpt4	[17]
pAM383	pCOLADuet-1 FLAG-Rpt1-Y283A, Rpt2, His_6-Rpt3, Rpt5-Q49TAG, Rpt6, Rpt4	This study
pAM384	pCOLADuet-1 FLAG-Rpt1, Rpt2-Y256A, His_6-Rpt3, Rpt5-Q49TAG, Rpt6, Rpt4	This study
pAM385	pCOLADuet-1 FLAG-Rpt1, Rpt2, His_6-Rpt3-Y246A, Rpt5-Q49TAG, Rpt6, Rpt4	This study
pAM386	pCOLADuet-1 FLAG-Rpt1, Rpt2, His_6-Rpt3, Rpt5-Q49TAG, Rpt6, Rpt4-Y256A	This study
pAM387	pCOLADuet-1 FLAG-Rpt1, Rpt2, His_6-Rpt3, Rpt5-Q49TAG-Y255A, Rpt6, Rpt4	This study
pAM388	pCOLADuet-1 FLAG-Rpt1, Rpt2, His_6-Rpt3, Rpt5-Q49TAG, Rpt6-Y222A, Rpt4	This study
pAM89	pCOLADuet-1 FLAG-Rpt1, Rpt2, His_6-Rpt3, Rpt5-Q49TAG, Rpt6, Rpt4	[17]
pAM81	pETDuet1 Rpn1, Rpn2, Rpn13	[11]
pAM83	pACYC-Duet RIL Nas6, Hsm3, Rpn14, Nas2	[11]
pAM87	pUltra_pAzFRS.2.t1_UAG-tRNA	[17]
pAM85	pETDuet1 Rpn5, MBP-HRV3C-Rpn6, Rpn8, Rpn11, Rpn9	[17]

pAM314	pETDuet1 Rpn5, MBP-HRV3C-Rpn6, Rpn8, Rpn11, Rpn9-F2TAG	[38]
pAM80	pACYC Sem1, Hsp90	[23]
pAM86	pCOLADuet-1 His_6-HRV3C-Rpn12, Rpn7, Rpn3	[17]
pAM239	pACYC Rpn10	[11]
pAM91	pCOLADuet-1 CBD-Titin I27 V15P (lysine-less)-PPPY-ssrA-1K-35 amino acid tail	[17]
pAM93	pCOLADuet-1 CBD-Titin I27 (lysine-less)-PPPY-ssrA-1K-35 amino acid tail	[17]
pAM395	pETDuet1 FLAG-Rpt1-Y283A	This study (derived from [11])
pAM396	pETDuet1 Rpt2 p1YA-Y256A	This study (derived from [11])
pAM397	pETDuet1 His-Rpt3-Y222A	This study (derived from [11])
pAM398	pETDuet1 Rpt4-Y246A	This study (derived from [11])
pAM399	pETDuet1 Rpt5-Y255A	This study (derived from [11])
pAM400	pETDuet1 Rpt6-Y255A	This study (derived from [11])
pAM403	pETDuet1 FLAG-Rpt1-I191TAG-Y283A	This study
pAM404	pETDuet1 Rpt5-Q49TAG-Y255A	This study
pAM431	pCOLADuet-1 FLAG-Rpt1, Rpt2, His_6-Rpt3, Rpt5-Q49TAG, Rpt6-E140A, Rpt4	This study
pAM432	pCOLADuet-1 FLAG-Rpt1, Rpt2, His_6-Rpt3, Rpt5-Q49TAG, Rpt6-K141A, Rpt4	This study

Supplementary Table 17. Plasmids utilized in this study (for sequences see Supplementary Data 1)

Yeast Strain	Phenotype / Expression	Source
yAM54	3xFLAG-Pre1	[11]
yAM80	Pre1-BirA-HRV-3xFLAG	[38]

Supplementary Table 18. Yeast strains utilized in this study
