# Supplementary material for: Substrate-interacting pore loops of two ATPase subunits determine the degradation efficiency of the 26S proteasome
Source: Nat Commun. 2026 Mar 24;17:4473. doi: 10.1038/s41467-026-70426-y (PMC13186983; doi:10.1038/s41467-026-70426-y)
Supplement: Supplementary file 2 — Description of Additional Supplementary Files [file 41467_2026_70426_MOESM2_ESM.pdf]

Description of Additional Supplementary Files

File name: Supplementary Data 1
Description: DNA sequences of the plasmids used for the recombinant expression of wild-type and mutant proteasome subunits, assembly factors, and model substrates used in this study.
