# Supplementary material for: Substrate-interacting pore loops of two ATPase subunits determine the degradation efficiency of the 26S proteasome
Source: Nat Commun. 2026 Mar 24;17:4473. doi: 10.1038/s41467-026-70426-y (PMC13186983; doi:10.1038/s41467-026-70426-y)
Supplement: Supplementary file 4 — Reporting Summary [file 41467_2026_70426_MOESM4_ESM.pdf]

nature portfolio

Corresponding author(s): Martin

Last updated by author(s): Jan 26, 2026

Reporting Summary

Nature Portfolio wishes to improve the reproducibility of the work that we publish. This form provides structure for consistency and transparency in reporting. For further information on Nature Portfolio policies, see our Editorial Policies and the Editorial Policy Checklist.

Statistics

For all statistical analyses, confirm that the following items are present in the figure legend, table legend, main text, or Methods section.

n/a	Confirmed	
☐	☒	The exact sample size (*n*) for each experimental group/condition, given as a discrete number and unit of measurement
☐	☒	A statement on whether measurements were taken from distinct samples or whether the same sample was measured repeatedly
☐	☒	The statistical test(s) used AND whether they are one- or two-sided<br>*Only common tests should be described solely by name; describe more complex techniques in the Methods section.*
☐	☐	A description of all covariates tested
☒	☐	A description of any assumptions or corrections, such as tests of normality and adjustment for multiple comparisons
☐	☒	A full description of the statistical parameters including central tendency (e.g. means) or other basic estimates (e.g. regression coefficient) AND variation (e.g. standard deviation) or associated estimates of uncertainty (e.g. confidence intervals)
☒	☐	For null hypothesis testing, the test statistic (e.g. *F*, *t*, *r*) with confidence intervals, effect sizes, degrees of freedom and *P* value noted<br>*Give P values as exact values whenever suitable.*
☐	☒	For Bayesian analysis, information on the choice of priors and Markov chain Monte Carlo settings
☒	☐	For hierarchical and complex designs, identification of the appropriate level for tests and full reporting of outcomes
☒	☐	Estimates of effect sizes (e.g. Cohen's *d*, Pearson's *r*), indicating how they were calculated

Our web collection on statistics for biologists contains articles on many of the points above.

Software and code

Policy information about availability of computer code

Data collection	For single-molecule FRET measurements, raw fluorescence images were aligned using a custom MATLAB script (https://github.com/jabard89/tirfexplorer) and loaded on Fiji to parse using the plugin Spot Intensity Analysis (https://imagej.net/plugins/spot-intensity-analysis).<br>For Cryo-EM, SerielEM was used for data collection.
Data analysis	For single-molecule measurements, fluorescence traces were sorted, plotted with Spartan software's built-in functions, and analyzed using hidden Markov modeling with ebFRET. In addition, a recently developed Bayesian nonparametrics (BNP) FRET-analysis algorithm, BNP-FRET, was used for data analysis, and the scripts for kinetic analysis are available through a GitHub repository at https://github.com/LabPresse/BNP-FRET-Binned.<br>CryoSPARC v4.4.0 was used for cryo-EM data analysis . Atomic models were rebuilt using Coot, and PHENIX was employed for docking and rebuilding.

For manuscripts utilizing custom algorithms or software that are central to the research but not yet described in published literature, software must be made available to editors and reviewers. We strongly encourage code deposition in a community repository (e.g. GitHub). See the Nature Portfolio guidelines for submitting code & software for further information.

Data

Policy information about availability of data

All manuscripts must include a data availability statement. This statement should provide the following information, where applicable:
- Accession codes, unique identifiers, or web links for publicly available datasets
- A description of any restrictions on data availability
- For clinical datasets or third party data, please ensure that the statement adheres to our policy

All data generated or analyzed during this study are included in this manuscript and the Supplementary materials. Structural data are available in the Electron Microscopy Databank and the RCSB Protein Databank (EMDB ID 45579 and PDB ID 9CGC for the non-processing, s1-state 26S proteasome). This paper does not report original code. Any additional information required to reanalyze the data reported in this paper is available from the lead contact upon request.

Research involving human participants, their data, or biological material

Policy information about studies with human participants or human data. See also policy information about sex, gender (identity/presentation), and sexual orientation and race, ethnicity and racism.

Reporting on sex and gender	NA
Reporting on race, ethnicity, or other socially relevant groupings	NA
Population characteristics	NA
Recruitment	NA
Ethics oversight	NA

Note that full information on the approval of the study protocol must also be provided in the manuscript.

Field-specific reporting

Please select the one below that is the best fit for your research. If you are not sure, read the appropriate sections before making your selection.

☒ Life sciences ☐ Behavioural & social sciences ☐ Ecological, evolutionary & environmental sciences

For a reference copy of the document with all sections, see nature.com/documents/nr-reporting-summary-flat.pdf

Life sciences study design

All studies must disclose on these points even when the disclosure is negative.

Sample size	For the analyses of single-molecule measurements, at least 50 traces or events were considered.
Data exclusions	For the single-molecule analyses, fluorescence traces that indicated static complexes or events of photobleaching were excluded.
Replication	Bulk measurements were performed with at least 3 technical replicates.
Randomization	N/A
Blinding	N/A

Reporting for specific materials, systems and methods

We require information from authors about some types of materials, experimental systems and methods used in many studies. Here, indicate whether each material, system or method listed is relevant to your study. If you are not sure if a list item applies to your research, read the appropriate section before selecting a response.

Materials & experimental systems

n/a	Involved in the study
☒	☐ Antibodies
☒	☐ Eukaryotic cell lines
☒	☐ Palaeontology and archaeology
☒	☐ Animals and other organisms
☒	☐ Clinical data
☒	☐ Dual use research of concern
☒	☐ Plants

Methods

n/a	Involved in the study
☒	☐ ChIP-seq
☒	☐ Flow cytometry
☒	☐ MRI-based neuroimaging

Plants

Seed stocks	NA
Novel plant genotypes	NA
Authentication	NA
